# Model-based design for seizure control by stimulation

**DOI:** 10.1088/1741-2552/ab7a4e

**Published:** 2020-03-26

**Authors:** Arian Ashourvan, Sérgio Pequito, Ankit N Khambhati, Fadi Mikhail, Steven N Baldassano, Kathryn A Davis, Timothy H Lucas, Jean M Vettel, Brian Litt, George J Pappas, Danielle S Bassett

**Affiliations:** 1 Department of Bioengineering, School of Engineering and Applied Science, University of Pennsylvania, Philadelphia, PA19104, United States of America; 2 Department of Electrical and Systems Engineering, School of Engineering and Applied Science, University of Pennsylvania, Philadelphia, PA 19104, United States of America; 3 Penn Center for Neuroengineering and Therapeutics, University of Pennsylvania, Philadelphia, PA 19104, United States of America; 4 Department of Neurology, Hospital of the University of Pennsylvania, Philadelphia, PA 19104, United States of America; 5 Department of Neurosurgery, Hospital of the University of Pennsylvania, Philadelphia, PA 19104, United States of America; 6 U.S. Army Research Laboratory, Aberdeen Proving Ground, MD 21005, United States of America; 7 Department of Psychological & Brain Sciences, University of California, Santa Barbara, CA 93106, United States of America; 8 Department of Industrial and Systems Engineering, Rensselaer Polytechnic Institute, Troy, NY 12180, United States of America; 9 Department of Psychiatry, Hospital of the University of Pennsylvania, Philadelphia, PA 19104, United States of America; 10 Department of Physics & Astronomy, College of Arts & Sciences, University of Pennsylvania, Philadelphia, PA 19104, United States of America; 11 Santa Fe Institute, Santa Fe, NM 87501, United States of America

**Keywords:** intracranial electrocorticography (iEEG), closed-loop stimulation, multivariate time-series analysis, dynamical stability analysis, eigenvalue-eigenvector structure

## Abstract

**Objective.:**

Current brain stimulation paradigms are largely empirical rather than theoretical. An opportunity exists to improve upon their modest effectiveness in closed-loop control strategies with the development of theoretically grounded, model-based designs.

**Approach.:**

Inspired by this need, here we couple experimental data and mathematical modeling with a control-theoretic strategy for seizure termination. We begin by exercising a dynamical systems approach to model seizures (n = 94) recorded using intracranial EEG (iEEG) from 21 patients with medication-resistant, localization-related epilepsy.

**Main results.:**

Although each patient’s seizures displayed unique spatial and temporal patterns, their evolution can be parsimoniously characterized by the same model form. Idiosyncracies of the model can inform individualized intervention strategies, specifically in iEEG samples with well-localized seizure onset zones. Temporal fluctuations in the spatial profiles of the oscillatory modes show that seizure onset marks a transition into a regime in which the underlying system supports prolonged rhythmic and focal activity. Based on these observations, we propose a control-theoretic strategy that aims to stabilize ictal activity using static output feedback for linear time-invariant switching systems. Finally, we demonstrate *in silico* that our proposed strategy allows us to dampen the emerging focal oscillatory sources using only a small set of electrodes.

**Significance.:**

Our integrative study informs the development of modulation and control algorithms for neurostimulation that could improve the effectiveness of implantable, closed-loop anti-epileptic devices.

## 1. Introduction

Understanding the dynamic neurophysiology that generates and propagates seizures is critical for preventing them, limiting their spread, and possibly arresting epileptogenesis[1].Perhaps the most immediate therapeutic application of this knowledge lies in the development of more effective implantable neurodevices [2], by informing patient-specific algorithms to modulate brain dynamics and abort seizures [3–5]. Ideally, these algorithms would dictate both *when* and *where* to deliver an intervention in the brain or connected structures. Effective stimulation protocols could improve quality of life for the nearly 1% of the world’s population affected by epilepsy [6], and dramatically for the 1/3 of these patients who are resistant to medication. Advances in stimulation are likely to come from engineering and control theory [7, 8], and it is surprising that little of it has been applied to current devices. This disconnect between theory and application is largely due to the relatively modest technical capabilities of early implantable devices, with their limited computational power, small channel count, and narrow recording bandwidth [2, 9]. However, these limitations are all being steadily overcome. Newer rechargeable versions of neurostimulation devices are currently in animal testing, and a host of established and newly formed companies, including Medtronic, NeuroPace, Neuralink, Blackfynn, and Neurotech Pharmaceuticals, Inc, are hard at work developing new, vastly more capable closed-loop devices for neural control and repair [10, 11]. Ultimately their success may critically depend on building a deeper understanding of the types of dynamical states that separate normal from pathological function.

A natural language in which to understand normal and pathological neural states is dynamical systems theory. Efforts to connect seizure physiology to dynamical systems theory are not new, and include work by Schindler *et al* and Rummel *et al*, who characterized the dynamical stability of the spatiotemporal correlation structure of intracranial EEG (iEEG) recordings over the peri–ictal period [8,12]. Modeling the dynamics of epileptic networks from iEEG is particularly challenging because epileptiform activity manifests across a broad range of spatiotemporal scales and displays marked inter-channel dependencies. Further challenges are posed by the tremendous patient heterogeneity in many features of the epilepsy syndrome and of the particular seizures[13–15]. Understanding specific biological phenotypes, therefore, requires the ability to assess interactions between different channels across different timescales, and generate testable causal models of seizure generation, propagation, and termination. Prior work demonstrates the utility of autoregressive models to identify seizures based on different features of system dynamics [16–18]. As an example, Franaszczuk and Bergey [18] argued that the goodness of fit of these models highlights the reduced complexity of the ictal onset regime, marking the transition from a less ordered state to a state of high regional synchronization. More recently, Mullen, Worrell [16] applied a multivariate eigendecomposition to analyze the time-varying principal oscillation patterns (or *eigenmodes*) of independent and spatially fixed sources of iEEG during two temporally proximate ictal samples. The group reported distinct shifts in characteristic frequency and damping time of a small subset of the most dynamically important eigenmodes before, during, and following seizures. Multivariate autoregressive models and related effective connectivity measures such as the adaptive directed transfer function (DTF) have also been used to model seizure propagation and improve identification of the seizure onset zone [19–26]. Moreover, it has also been shown that similar linear models can be successfully deployed to explain and predict the response of brain tissue to direct external electrical stimulation [27].

A key challenge facing these and other studies that attempt to use dynamical systems theory to better understand seizure activity in humans is the marked variability in clinical presentation. Here we address this challenge by examining data from a particular subset of epilepsy patients who present challenges to current medical practice. Specifically, we chose individuals with medically refractory localization-related epilepsy of neocortical origin, who underwent presurgical evaluation with intracranial EEG recordings. These patients are typically implanted with intracranial electrodes in tertiary epilepsy centers prior to surgical intervention, but have, on average, a 40% chance of seizure freedom with surgery. This is a group that is most appropriate for treatment with implanted neurostimulation devices, which offer great potential for therapeutic benefit with much lower morbidity than resective or ablative surgery.

Although seizure onsets are qualitatively different across patients, we will demonstrate that ictal onset activity in our patient sample manifests as the emergence of prolonged focal oscillatory eigenmodes in seizure samples with good localization of the seizure onset zone(SOZ).In fact, prior evidence suggests that the presence of observed low-voltage high-frequency activity(LVHF),and sharp rhythmic activity are some of the most reliable hallmarks of the SOZ [28, 29]. Importantly, recent evidence suggests that oscillations in the local field potentials (LFPs) captured by iEEG electrodes following seizure onset, are not only a sign of synchronized activity of the neural population at the ictal core, but also causally contribute to synchronized population activity via ephaptic coupling (for a review see [30]). Together, these observations suggest that disrupting LFP oscillations following the seizure onset provides a viable target for seizure intervention. We leverage this fact in positing a dynamical systems model and associated seizure control strategy that is deployable prospectively, in real-time epilepsy control devices.

Recently, we developed a closed-loop actuation strategy for static output feedback control for linear time-invariant switching systems [31]. Based on our preliminary results as well as other reports of anomalous excursions of frequency and dynamical stability (i.e. dampening) of the oscillatory eigenmodes [16], we conjectured that spectral control of cortical activity is a promising early seizure intervention strategy [31]. One of the limitations of our spectral control algorithm is that it is agnostic to changes in the spatial profile of the underlying system. Nevertheless, its success hinges heavily on our ability to accurately model the epileptic sources. In this paper, we explicitly model the system in a manner that directly captures the interplay between brain regions sampled by different electrodes during the peri–ictal period. Specifically, we leverage the information contained in the contribution of different channels at spatiotemporal frequencies that reflect seizure dynamics using multivariate autoregression. We demonstrate that the oscillatory modes of the system change in their spatial profiles in a manner that is time-locked to spectral fluctuations occurring at seizure onset, specifically for those seizures that have a clearly localized focus. These results suggest that information regarding the spatial profile of the system and its temporal fluctuations could inform seizure intervention strategies both spatially and temporally in real-time.

Building on the evidence provided by our observations, we make the case that the ictal onset regime provides a critical window for deploying our proposed spectral control algorithm [31] to dampen focal oscillations in a targeted manner. The theory predicts that external control of the LFP could terminate the seizure by disrupting the electrical field oscillations that causally drive the synchronization at the ictal core through ephaptic coupling [30] or alternatively, reduce the likelihood of seizure spread by focal dampening. Finally and with the aim of offering a proof-of-principle demonstration, we provide synthetic examples modeled from the iEEG to show that the static feedback gains afforded by our strategy can successfully increase the damping rate of focal ictal oscillations in an ideal closed-loop switching system. Broadly, our approach provides a compact mathematical representation that can be quickly quantified and optimized for stimulation control, which can inform the development of closed-loop implantable therapeutic devices [32–35].

## 2. Materials and methods

### 2.1. Description of experimental data

In this study, we analyzed recordings from twenty-one subjects undergoing presurgical evaluation for medically refractory epilepsy of neocortical origin. These subjects underwent implantation of subdural electrodes to localize their epileptic networks after a noninvasive presurgical evaluation suggested a focal, surgically amenable epilepsy. The evaluation was performed in accordance with the National Association of Epilepsy Centers (NAEC) standards, with video-scalp EEG acquired in the epilepsy monitoring unit (EMU), high resolution structural and functional MRI, positron emission tomography (PET), ictal single photon emission tomography (SPECT), and neuropsychological testing. All data had previously been de-identified and made publicly available via the International Epilepsy Electrophysiology Portal (IEEG Portal) [36]. Patients exhibited a broad range of etiology, type, and severity of epilepsy, providing a rich, representative dataset for assessing seizure dynamics in this population. Table 1 describes the clinical characteristics of these subjects, who were evaluated at the Hospital of the University of Pennsylvania and the Mayo Clinic, Rochester.

### 2.2. Intracranial electroencephalography (iEEG) recordings

iEEG signals were recorded and digitized at sampling rates of 512 Hz (Hospital of the University of Pennsylvania, Philadelphia, PA) and 500 Hz (Mayo Clinic, Rochester, MN). Subdural grid and strip electrodes (Ad Tech Medical Instruments, Racine, WI) were placed in locations that were determined by a multidisciplinary team of neurologists, neurosurgeons, and radiologist. Electrodes consisted of linear and two-dimensional arrays spanning 2.3 mm in diameter with 10 mm inter-contact spacing. Signals were recorded using a referential montage with the reference electrode chosen by the clinical team to be distant to the site of seizure-onset. Recordings spanned the duration of a patient’s stay in the epilepsy monitoring unit (range: 15 d).

A total of 94 seizures were marked by clinical experts (see [37]) and the seizure-onset time and localization were defined by the points of ‘earliest electrographic change’ (EEC) and ‘unequivocal electrographic onset’ (UEO) [38]. Using the EEC, we extracted multi-channel iEEG time series from seizure onset to seizure termination, with a buffer of 20 s on each side of this time period. Lastly, we extracted a total of 478 (22.7 ± 7.3 segments per patient) randomly selected inter-ictal segments (lasting 100 s each)fromtracesacquiredmorethan3hbeforeor3h after a seizure. Time series were high-pass filtered to maintain information in the frequency range>0.1Hz.

### 2.3. Clinical assessment of the seizure onset zone and localizing patients

Seizure-onset Zone (SOZ) electrodes are identified clinically on the IEEG according to the standard clinical protocol in the Penn Epilepsy Center and Mayo Clinic. The SOZ markings are initially made by a board-certified epileptologist on the day of each seizure, and vetted and updated weekly at the surgical conference according to a consensus marking of four board-certified epileptologists. Finally, SOZ markings on IEEG are then assessed in the context of other tests such as MRI, PET scan, MEG, single-phone emission computed tomography, and neuropsychological testing for surgical planning. This is the standard process of clinical care for epileptic patients in the United States at Level-4 epilepsy centers certified by National Association of Epilepsy Centers. Additionally, patients are further divided into two groups based on the quality of SOZ localization following the clinical assessment of the iEEG by a board-certified epileptologist (F.M).

### 2.4. Dynamical stability approach

The iEEG signals can be locally approximated at each time point by a linear system [39]. Let *x*(*k*) ∈ *R*^*n*^ be the data from *n* channels, where the *i*-th entry *x*_*i*_ (*k*) corresponds to the data collected by channel *i* at time *k*. Then, we obtain

(1) x(k)=Ax(k−1)+ε(k)

where *A* is the *n* × *n* real matrix that results from fitting the data collected within the interval of time [*k* − τ, *k* + τ] using a least-squares approach, and where ε(*k*) is the approximation error. As a consequence of the linear approximation, the results will depend on the choice of the number *n* of channels and the size of the time window parameterized by τ. In the Results and supplementary materials, we further discuss the impact of these parameters on the model.

A significant advantage of using a linear approximation to model the dynamics is that the dynamical properties of the underlying process can be locally assessed through the eigendecomposition of the matrix *A*. The *n* eigenvalue-eigenvector pairs capture linearly independent spatiotemporal dynamical processes. Specifically, for each putative spatiotemporal process, the eigenvector weights the involvement of each sensor and the complex eigenvalue defines the frequency of the oscillatory dynamics. More importantly, the stability of the dynamics can be captured by the absolute value of the eigenvalues, which forecast the exponential growth or decay along the associated eigenvector.

Specifically, let *A* = *VλV*^*T*^ be the eigendecomposition for a given time point *k*, where *V* = [*v*_1_*, ..., v*_*n*_] and *λ* = *diag*(*λ*_1_*,..., λ*_*n*_) are the matrices of eigenvectors and eigenvalues, respectively. The pair (*λ*_*i*_*, v*_*i*_) is an eigenvalue paired with the associated eigenvector, and *V*^*T*^ is the transpose of *V*. Notice that some eigenvalues are complex numbers, which implies that no partial-order can be imposed on the eigendecomposition. Further, note that after *T* time steps, one obtains from equation (1) that *x*(*k*) = *A*^*T*^*x*(0), which implies that *x*(*k*) = *VλV*^*T*^*x*(0). Subsequently, by considering a linear combination of the original data *z*(*k*) = *V*^∗^*x*(*k*), where <di>*z*_*i*_ (*k*) = *v*^*T*^_*i*_ *x*(*k*) $z_{i}(k)=v_{i}^{T}x(k)$ is a weighted combination described by the *i*-th eigenvector associated with the *i*-th eigenvalue.

From these definitions, it follows that $z_{i}(k)={\left|\lambda_{i}\right|}^{T}\left|z_{i}(0)\right|$, and three scenarios are possible: (*I*) |*λ*_*i*_| *<* 1 which leads to $\left|z_{i}(k)\right|\to 0$ as t→∞; (ii) $\left|\lambda_{i}\right|>1$ which implies that $\left|z_{i}(k)\right|\to \infty \;$ as t→∞; and $\left|\lambda_{i}\right|=1$ where $z_{i}(k)=\left|z_{i}(0)\right|$ for all times. In (*i*), the process tends to vanish, and we, therefore, refer to these dynamics as *asymptotically stable*. In (*ii*), the process tends to explode, and we, therefore, refer to these dynamics as *unstable*. Finally, in (*iii*), the process oscillates between stability and instability, and we, therefore, refer to these dynamics as *stable*. In practice, we consider that the stable regime is determined by |*λ*_*i*_| ≈ 1. Since these processes are associated with *z*(*k*), it follows that they are associated with a specific eigenvector, and, consequently, we refer to a stable regime associated with a particular eigenvector. Thus, the interplay between these three different stages and different eigenvectors provides a *dynamical stability* characterization to identify seizure onset and to monitor seizure evolution.

To further characterize these dynamics, we note that the angle θ_*i*_ associated with the polar coordinates of the *i*-th complex eigenvalue describes the *frequency* as follows:

$$f_{i}=\frac{\theta_{i}}{2\pi }\delta_{t}$$

where δ*t* corresponds to the sampling frequency. The *timescale* is given by

$$\rho_{i}=\frac{\log\left(\left|\lambda_{i}\right|\right)}{\delta_{t}}$$

which can be interpreted as the growth rate. Therefore, the dynamical process *z*(*k*) describes the spatiotemporal behavior of the dynamical system, where the timescale is encoded in the eigenvalue and the spatial scale is encoded in the eigenvector, indicating the relative contribution of a given channel or—by extension—the overall activity in a specific cortical region.

We use this approach to consider the time-evolution of eigenvalue-eigenvector pairs associated with higher and lower frequencies, and associated with fine and coarse timescales, to better understand seizure onset, propagation, and termination. In the supplementary materials, we define and study synthetic examples of damping *versus* growing oscillations to demonstrate how dynamical stability analysis quantitatively characterizes the underlying process.

### 2.5. Analysis pipeline

In iEEG recordings obtained from twenty-one patients with medically refractory neocortical epilepsy, we used dynamical stability analysis to estimate equation (1) over a 1 s sliding-window for 100 ms shifts. Specifically, we estimated the matrices **A** using previously developed computational algorithms [40], and then we calculated the eigenmodes (i.e. the eigenvalue-eigenvector pairs) of the estimated **A** matrices for each time point *k*. The eigenvectors are considered based on their spatiotemporal frequency; that is, the frequency determined by the corresponding eigenvalue. Because the eigenvector may contain several non-zero entries, the frequency of the dynamic process along the direction determined by the eigenvector does not coincide with any specific iEEG channel. Instead, it corresponds to the spatiotemporal frequency along the direction captured by the eigenvector. For ease of visualization, we normalize the eigenvectors by dividing each entry by the maximum value obtained across the entries, and then we take the absolute value. Thus, all entries correspond to values between 0 and 1, and capture the contribution of different iEEG channels to the evolution of the underlying neurophysiological process at a certain spatiotemporal frequency.

### 2.6. Statistical testing

Because the distributions of our statistics of interest were not known *a priori*, we used a non-parametric Wilcoxon rank-sum test to assess the statistical significance of the fluctuations in the maximum values as well as the kurtosis of the distributions of eigenvector loadings before and after the seizure onset (see supplementary figure 2 (stacks.iop.org/JNE/17/026009/mmedia)). Similarly, we used a Wilcoxon rank-sum test to assess the statistical significance of the reduced loading of SOZ electrodes in the closed system versus the open system at ictal onset in figures 4 and 5.

### 2.7. Generating synthetic time series

**Figure 1.**
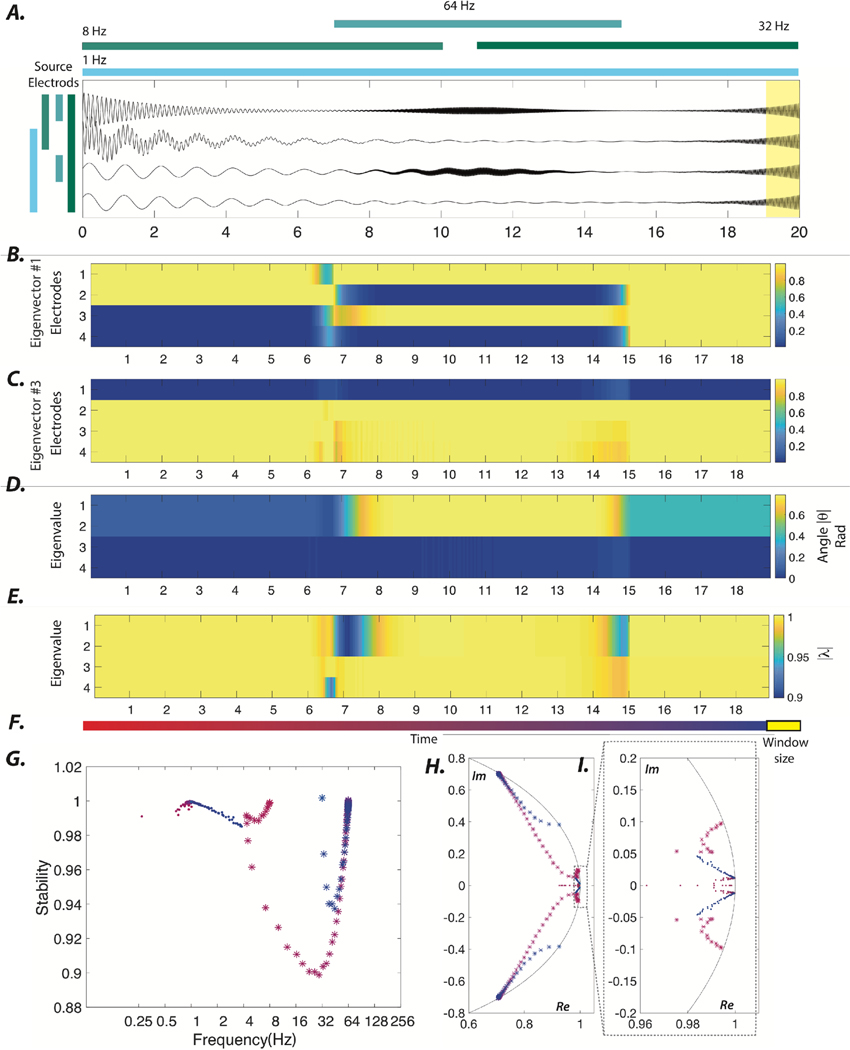
Dynamical stability of synthetic time series. (A) To gain intuition, we constructed synthetic time series sampled at 512 Hz that capture an underlying emerging process defined by an enveloped (exponential, Gaussian) sinusoid. Briefly, the simulated time series captures various partially overlapping spatiotemporal patterns of oscillations with varying frequencies. The colored bar at the top of the panel represents the duration over which the oscillation with that specific frequency is present. The colored bars on the left code the sources of the oscillations, where the top source leads the following source by $\frac{\pi }{2}$. The yellow shaded area on the right indicates the size of the moving window. (B), (C) The absolute values of the normalized eigenvectors 1 and 3 obtained using a 1 s window shifted by 100 ms intervals. (D), (E) The evolution of the angle (reflecting the oscillation frequency) and radius (reflecting the stability) associated with the four eigenvectors of the 4-sensor system. Note that the changes in the angle and radius associated with higher frequency eigenmodes (i.e. eigenmodes 1 and 2) between the 6 and 8 s timepoints mark the transition and emergence of high frequency (64 Hz) oscillations between electrodes 1 and 3. (F) The temporal progression of the sliding window illustrated in changing hues of red; the yellow box represents the relative size of the moving window used to estimate the linear model. (G) The temporal evolution of the frequency (Hz) and stability associated with the first eigenmode (‘dot’) and third eigenmode (‘star’). (H) The temporal evolution of the four eigenvalues in the Argand complex plane. (I) A zoomed-in version of the panel (H) is provided to show the changes in the angle and radius of the low angle eigenvalues in greater detail. Panels (G)–(I) use the same color-coding as in panel (F). In sum, this example demonstrates how the dynamical properties of oscillatory sources that are spatiotemporally overlapping are reflected in the eigenvalue-eigenvector pairs and their fluctuations over time.

In the first section of the Results, we provide a pedagogical example of the dynamical stability-based approach and demonstrate its ability to accurately uncover a planted locus, frequency, and damping of oscillatory dynamics. Here we provide additional information about this synthetic time series. Specifically, the synthetic dataset that we study consists of four channels associated with four oscillatory sources sampled at 512 Hz. Each source is modeled as an enveloped (exponential, Gaussian) sinusoid, and the four sources have a fixed lag between them to induce directionality of information flow across sensors. Specifically, the baseline activity of each channel is modeled as follows:

$$\sin(ax+b)e^{cx},$$

where *a* determines the frequency of the oscillation, *b* captures the phase lag between electrodes, and *c* determines the damping of the oscillations. Negative values of *c* were used for early damping oscillations while positive values of *c* were used for late-emerging high-frequency oscillations. In addition to this baseline activity, we also modeled transient activity that emerges and disappears in brief intervals during the recordings. We model these transient dynamics as follows:

$$y=\sin(ax+b)\frac{1}{\sqrt{2\sigma^{2}\pi }}e^{-\frac{\left(x-\mu \right)}{2\sigma^{2}}}$$

where *μ* is the mean and *σ* the standard deviation of a normal distribution. Finally, different simulated patterns of oscillatory activity over time were linearly superimposed to create the activity of individual channels. Additional details regarding this process of sensor time series creation are depicted schematically in figure 1(A).

## 3. Results

### 3.1. Dynamical stability analysis of synthetic time series

Here we provide a synthetic example of the dynamical stability-based approach, and demonstrate its ability to accurately uncover the planted locus, frequency, and damping rates of oscillatory dynamics (figure 1(A)). The synthetic dataset that we study consists of four channels with four spatiotemporally distributed non-stationary oscillatory sources (for more details see section 2). First, we show the eigenvector evolution associated with the highest frequency dynamics in the signals (figure 1(B)), and then we show the eigenvector evolution associated with dynamics in a lower frequency (figure 1(C)). We observe that the locus of higher and lower frequency oscillations at every time point can be identified via electrodes with relatively higher values in the eigenvectors 1 and 3, respectively. We also display the stability, |λ| associated with those same two eigenvectors (figures 1(D), (E)). We notice that the changes in the angle match the changes of the underlying frequency of oscillations, while the intervals with radius (> 1) highlight periods of instability (captured at the very last estimation window). Lastly, we studied the temporal evolution (figure 1(F)) of the statistics of interest derived from the eigenmode decomposition (figures 1(G)–(I)). Together, these results demonstrate that the dynamic stability approach is able to accurately uncover the planted structure of the oscillatory dynamics and that an examination of the time-evolution of eigenvalue-eigenvector pairs allows veridical characterization of the spatial profiles, damping rates, and frequencies of underlying processes. In the following section, we demonstrate how the dynamical stability-based approach reveals the emergence of epileptic sources at the ictal onset.

### 3.2. Dynamical stability analysis pinpoints seizure onset by identifying distinct spatiotemporal patterns

Successful localization of the seizure onset zone and the epileptic network from iEEG recordings is foundational to our ability to model and ultimately control the underlying seizure dynamics. After clinical assessment of the iEEG by a board-certified epileptologist (F.M), we divide seizures into ones with and without a clear localization of the SOZ. We use previously validated and published localizing features to distinguish between the two groups, as well as postsurgical seizure freedom. iEEG features that allow us to localize the seizure onset include (i) the presence of low voltage fast activity (LVFA), (ii) the presence of ultraslow transient polarizing shifts, and (iii) voltage attenuation or rapid spiking activity [41]. If these features are not observed, we refer to the dataset as nonlocalizing; such datasets commonly exhibit a pattern typical of seizures propagated from elsewhere in the brain, usually marked by the presence of slow delta activity often involving a large number of electrodes or slow spiking activity, as shown in supplementary figure 1. For the remainder of this study, we restrict our attention to seizures with a clear localization of the SOZ, which we observed in 10 (out of 21) patients (see table 1).

After defining the seizures of interest, we performed dynamic stability analysis. Recall that our synthetic example demonstrated that emerging local oscillatory sources are captured by the evolution of eigenvalue-eigenvector pairs. We hypothesize that the emergence of focal epileptic sources following ictal onset are similarly captured by eigenmodes of the system, which we can identify using a sliding-window approach. We anticipate that transitions of the system to distinct phases of the seizure will be time-locked to changes in (i) the *frequency,* which is given by the angle associated with the eigenvalues, (ii) the *stability,* which is measured by the radius associated with the eigenvalues, and (iii) the spatial profile, which is given by the eigenvectors in the eigenvalue-eigenvector pairs.

**Figure 2.**
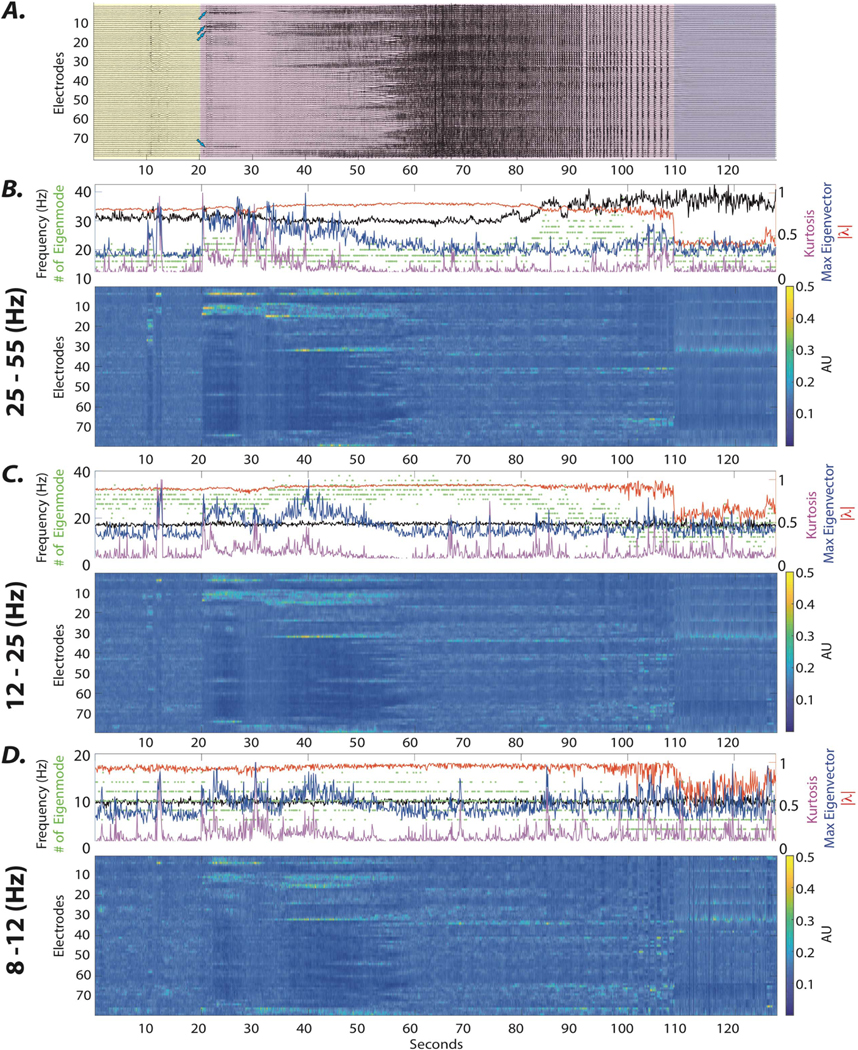
The evolution of the eigenmodes over the ictal period. (A) Here we show iEEG signals from 79 electrodes in subject 3 over a single seizure, beginning 20 sec before the seizure onset and ending 20 sec after seizure termination. The pre-ictal activity (yellow), ictal period (purple), and post-ictal period (gray) are delineated separately. (B–D) The heat map (bottom) demonstrates the mean absolute value of eigenvectors associated with γ, β, and α band frequencies, respectively. Notice that the emergence of ictal sources is marked by high and focal loading on SOZ electrodes across all frequencies. We quantify these changes in the distributions of eigenvector loadings by providing the maximum values (blue line) and kurtosis (purple line) of these distributions in the top panels. Notice that the increase in these values is time-locked to the seizure onset across frequencies; we find this phenomenon to be a common property across all focal localizing seizure samples (supplementary figure 2). The emergence of focal sources is also reflected in the fluctuations of the number (green dots), the stability (red line), and the frequency (black line) of the higher frequency eigenvalues time-locked to the seizure onset. Broadly, this example seizure and the quantitative characterization of the same features across all localized seizures (supplementary figure 2) together demonstrate that the identified systems capture the emerging ictal sources and allow characterization of properties of the underlying dynamics.

We now turn to test our hypothesis and expectations. To provide the reader with greater intuition, we consider a representative focal seizure from the group of patients with clear localization of the SOZ (see figure 2(A)). Next, we demonstrate that the changes in the stability and frequency of system eigenmodes estimated using a one-second sliding window accurately capture the onset and spatiotemporal evolution of the seizure(see figures 2(B)–(D)).Nevertheless, the change in the spatial profile of the eigenvectors of the system following the ictal onset is the most salient feature of the seizure onset across localizing samples. We observe that the transition to the regime of focal oscillations at the onset of the seizure is marked by an increase in the loading of eigenvector elements corresponding to the SOZ electrodes. We quantified the changes at seizure onset via the maximum value and kurtosis of the eigenvectors (see figures 2(B)–(D)). We observe a significant wide-band increase in the maximum value and kurtosis of the eigenvectors (supplementary figure 2, Wilxocon rank-sum test, *p* < 0.05), reflecting the emergence of focal ictal sources in patients with localizing iEEG recordings. In the following section, we provide additional evidence supporting the notion that dynamical stability analysis, in theory, can be leveraged to inform individualized seizure intervention algorithms.

In this work, we chose a first-order LTI to linearize each nonlinear dynamical system. If the system trajectory remains close to the equilibrium point or to the nominal trajectory, then the first-order linear model is close to the real nonlinear system. Then based on the linear model, one can design a linear dynamic output feedback controller to regulate the dynamics [42]. In practice, in either linear or nonlinear dynamical system, we should expect measurement errors. These errors can arise from perturbation of the system by additive stochastic noise [43], and/or by the presence of recording noise.

In order to improve the fit of the LTI model, one can increase the order of the model and tests like Akaike information criterion (AIC) or Bayesian information criterion (BIC) can be used to identify the number of lags necessary for an accurate fit. We show a higher-order (n = 2) fit to an example seizure onset to evaluate the robustness of the characterized ictal behavior to increases in the order of the model (supplementary figure 5). Nevertheless, to examine the goodness-of-fit of the first-order LTI model, we calculate the average estimated order of the model in a 5-second window before and after the unequivocal onset of all seizure samples from localizing patients. Supplementary figure 6 demonstrates that both criteriayieldaveragemodelordershigherthan1,regardless of the spatiotemporal sampling (supplementary figures 6(A) and (B)), although with a high degree of variance (supplementary figures 6(C) and (D)). These results suggest that we should expect a degree of error in our estimated first-order LTI parameters; however, because the theory is more tractable in the first-order case, we constrain ourselves to an investigation of AR1 in this paper, and leave an extension of the theory to AR2 for a future study.

Next, we provide a systematic investigation of the dependence of the estimated system on the sampling rates and the number of recorded areas over the peri–ictal period. Supplementary figures 7–10 show how variations in the spatial and temporal sampling impact the band-passed average kurtosis of the eigenvectors, the maximum value of eigenvector elements, frequency, and stability of the eigenvalues estimated from a window before and after the onsets of all seizure samples from localizing patients. Notably, the significant changes in the kurtosis and maximum value of eigenvector elements following the unequivocal seizure onsets over higher frequency bands are relatively robust, being identified with sampling frequencies as low as 125 Hz and using as few as 25 randomly sampled electrodes. These results also show the importance of the number of electrodes and effective coverage of seizure sources; the modeled systems using only a few (e.g. n = 5) randomly sampled electrodes fail to accurately capture the rich multi-scale dynamics of seizures.

These results are best highlighted in the average estimated frequency and stability of the eigenvalues in supplementary figures9–10. Note that for instance, the estimated systems from 5 electrodes at higher sampling rates do not contain eigenvalues associated with beta (12–25 Hz) or gamma (25–55 Hz) frequency bands. Intuitively, these results demonstrate the dependence of the estimated system to spatiotemporal sampling and suggest that greater spatial and temporal sampling of ictal sources enhances our ability to accurately model the seizure onset oscillations.

### 3.3. Generalized pole placement offers potential solutions for stabilizing seizure onset dynamics

In the previous sections, we presented a model-based approach to characterize different events of interest in a dynamical system and then an application of that approach to seizures to measure the frequency, stability, and spatial profile of coherent oscillations. While of interest, more important is the opportunity to use this method to guide interventions to control seizure dynamics. We have established how eigenmode properties capture the evolution of the well-localized epileptic network during the transition from a disordered pre-ictal regime—characterized by activity among different time-variant cortical regions—to a highly ordered ictal regime—characterized by the emergence of focal oscillatory sources (supplementary figure 2). Therefore, we hypothesize that by properly crafting neurostimulation parameters, one might be able to constrain the evolution of the seizure, or alternatively, to stabilize (i.e. increase the damping rate of) the persistent oscillations that follow the seizure onset.

Specifically, we pose the problem as one of determining the static output feedback [44] that jointly ensures spectral properties across the different modes of the system. Static output feedback entails injecting a quantity proportional to the collected measurements of the system’s evolution. Such a strategy has the advantage of not requiring the response to a given output signal to be computed at each time point, which might be prohibitive for implantable neurodevices, which have low computational power and limited energy resources. In the context of neurostimulation, most of the strategies employed to date are open-loop, and only recently has the field begun to consider closed-loop proportional-integral-derivative (PID) strategies [45–48].

**Figure 3.**
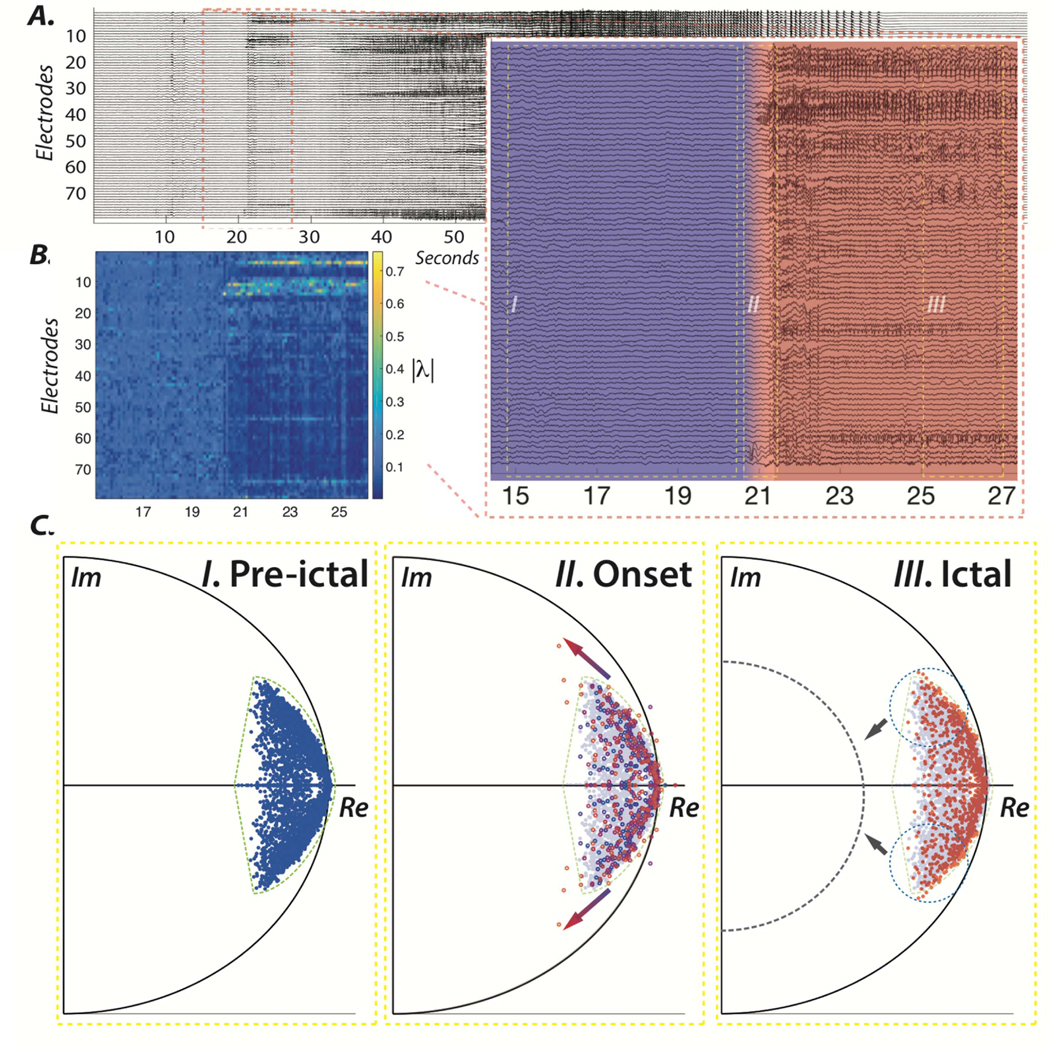
Regulation of ictal activity using a dynamical stability control strategy. (A) iEEG signals from 79 electrodes in subject 3 over a single ictal period, beginning 20 s before seizure onset and ending 20 s after seizure termination, as shown previously in figure 1. The inset on the bottom right shows the emergence of epileptic activity and the transition from pre-ictal (blue) to ictal (red) dynamics following the seizure onset. (B) The evolution of the absolute value of the third eigenvector associated with high frequencies (mean = ~ 38 Hz, std = 2.86) displayed in the heat map. (C) Argand diagrams display the position of the eigenvalues over the course of (I) pre-ictal, (II) onset, and (III) ictal periods. Ictal onset is marked by the excursion of several eigenvalues from the pre-ictal zone (dashed green). An early intervention control strategy could entail closed-loop actuation, with the aim of driving the eigenvalues of the system closer to the center, towards the asymptotically stable zone (dashed gray) and, thus, in effect damping the focal epileptic dynamics via field effects.

Following this line of work, we recently described a static output feedback control methodology to regulate the evolution of eigenvalues at the seizure onset. Figure 3(C) panel II shows an example of the excursions of eigenvalues from their per-ictal location during the onset of a sample seizure. However, as reported in the previous sections, both eigenvalues and eigenvectors may prove useful in the design of seizure control strategies. Unfortunately, to find such a closed-loop solution is computationally intractable, but the challenge can be overcome by decomposing the original problem in two sub-problems, where one can be exactly and efficiently solved, and the other can be approximated in a least-squares-like sense that can also be efficiently determined. We provide additional technical details regarding these problems and the generalized pole placement algorithm in the supplementary information section. Building on the insights obtained from the previously described methodology, we propose an alternative strategy and control objective based on stabilizing focal seizure activity in a period immediately following the seizure onset using static output feedback. The schematic in figure 3(C) panel III shows how the proposed control law could moderate the evolution of the seizure following its onset by ensuring that the poles (i.e. eigenvalues) of the closed-loop system are placed in a prespecified location that only supports stable dynamics.

In the previous sections, we demonstrated that dynamical stability analysis allows us to characterize the spatial and spectral properties of the ictal onset oscillations. Although our results show that the ictal onset regime is marked by emerging ictal sources that can last over several seconds, yet we expect the estimated system parameters to fluctuate over time. These fluctuations may reflect the slow changes in the underlying seizure sources, in addition to artifacts due to the presence of recording noise, system identification error, and temporally overlapping non-stationary oscillations (as seen in the synthetic example in figure 1). From a seizure intervention standpoint, system switches can significantly limit our ability for real-time control using static output feedback and pole placement algorithms (e.g. [49, 50]). Pole placement algorithm is a numerical method for determining well-conditioned solutions to the problem of pole assignment by state feedback [49]. However, the calculated feedback gains only guarantee results for a single window of estimation. To address this limitation, we leverage the method proposed in [31] that enables a generalized pole placement method for spectral control of switching linear systems. Here, we provide a computational simulation to demonstrate how our proposed intervention strategy and generalized pole placement may allow us to stabilize oscillations at the ictal onset by increasing their damping rate.

**Figure 4.**
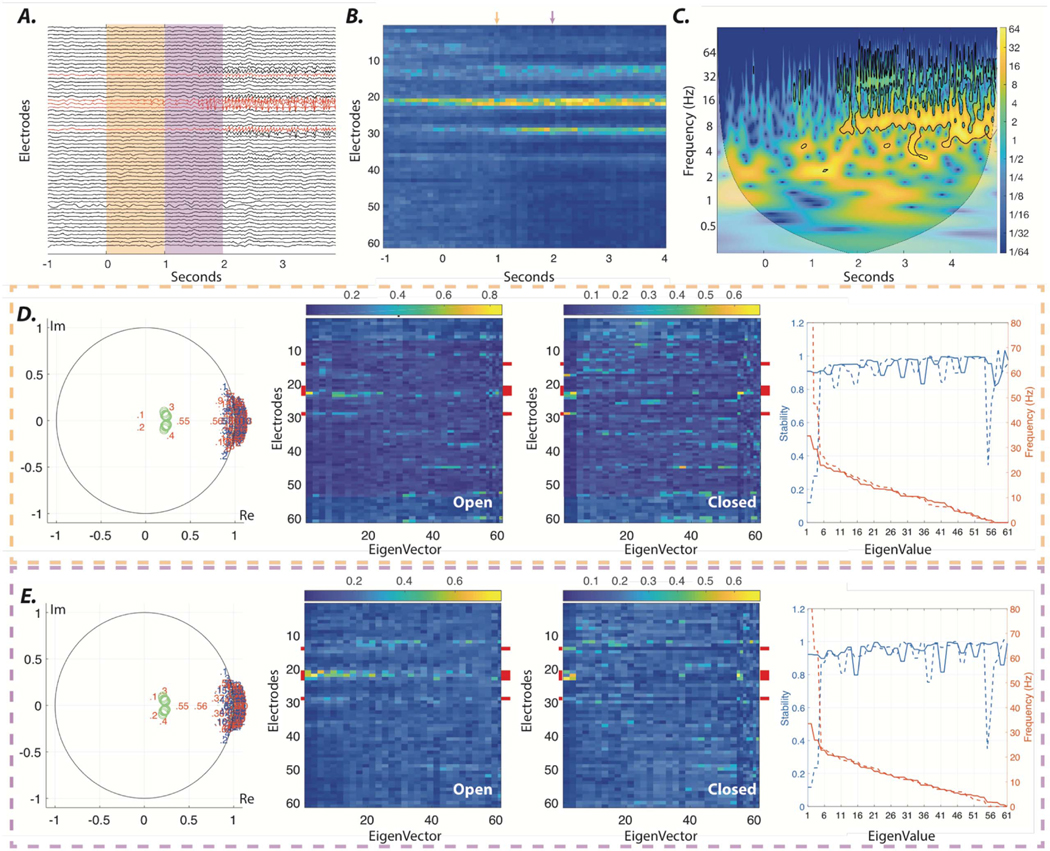
Stabilizing ictal onset oscillations using closed-loop feedback control. (A) Seizure onset sampled from a patient with iEEG recordings from which we can clearly localize the SOZ. (B) Mean absolute values of all eigenvectors, whose associated eigenvalues reside at β- and γ—band frequencies (15–50 Hz) across the 1-sec sliding windows. The orange and purple arrows highlight the high loading of a few electrodes in two selected windows at the seizure onset presented in panel A. (C) Wavelet decomposition of a SOZ electrode highlights the emergence of oscillations at high frequencies following seizure onset. (D) The left panel shows the distribution of the estimated eigenvalues at seizure onset (orange window in panel A). Eigenvalues are sorted based on their frequencies, from highest to lowest. Blue numbered dots represent the empirically estimated values. We simulate the effect of closed-loop static feedback between a few electrodes (marked by red in panel A), by representing eigenvalues of the closed system (red numbered dots). Only five electrodes with the highest eigenvector loading at seizure onset (as seen in panel B) were selected as sensing and stimulating electrodes to mimic the limited channels of implantable neurostimulation devices. The static output feedback gains were calculated using the generalized pole placement method [31], with the control-theoretic objective of shifting all the higher frequency (>15 Hz) eigenvalues of systems that were estimated from ten consecutive sliding widows following the seizure onset to the predefined zones, represented by green circle (for more details see supplementary information). The two middle panels show the absolute values of eigenvectors of the open and closed systems. The right panel shows the stability (i.e. absolute values of eigenvalues) and frequency of all eigenvalues in both open (solid lines) and closed (dashed lines) systems. Note the reduced stability of all the closed system eigenvalues with associated eigenvectors with high loading on stimulating electrodes (marked by red in middle panels); namely, eigenmodes 1–4 (See supplementary figure 4 for statistical test results). (E) Same results as those represented in panel D, calculated for the purple window in panel A. Together, this demonstration suggest that the calculated static feedback can similarly stabilize the seizure activity across all seizure onset windows.

To gain an intuition for how such an intervention would work, we first show the onset of a seizure from a sample patient with localizing iEEG (figure 4(A)). We identify system parameters using a one-second sliding window. Similar to the synthetic example in figure 1, the evolution of the identified system’s eigenvectors highlights the focus and onset of the seizure (figure 4(B)). We propose to increase the stability of these emerging high-frequency oscillations by providing static output feedback. In supplementary figure 3(A), we demonstrate that the feedback gains calculated at the onset of the sample seizure in figure 4(A) using pole placement method [49] successfully shifts all the higher frequency eigenvalues (> 15 Hz) of the closed system corresponding to emerging ictal oscillations, to the desired stable zone only by using a few (n = 5) stimulation loci. We selected a small set of electrodes with the highest eigenvector loading at seizure onset as the stimulating electrodes. Despite the apparent local success of the pole placement method, we note that this algorithm requires the feedback gains to be calculated for all electrodes, which can be limiting in real-world applications. Further, we note that the pole placement method, unfortunately, fails globally to stabilize the emerging ictal dynamics even after one second from seizure onset (supplementary figure 3(B)).

To address these limitations, we explored the scenario in which only a few sensing and stimulating electrodes are available (n = 5) and sought to determine whether the feedback gains that were calculated using our proposed generalized pole placement method from several consecutive sliding windows are able to reduce the stability of the ictal oscillations. We observe that despite the number of sensing and stimulating electrodes constraining the number of the eigenvalues that are shifted to the stable zone, the stability of seizure onset oscillations is effectively reduced (figure 4(D)). More importantly, we observe that the calculated feedback gains similarly allow us to reduce the stability of ictal oscillations over time, despite the continued evolution of system parameters (figure 4(E)). Collectively, these numerical experiments suggest that our method allows us, in theory, to prevent the spread and evolution of focal seizures by early dampening of ictal activity.

Here we focused on patients with localizing iEEG samples for two reasons. First, the non-localizing iEEG samples do not offer clear surgical targets for electrode placement, and consequently, these patients are rarely implanted with neurostimulation devices. Second, our approach requires the following mechanism: that damping the SOZ’s activity affects the activity of surrounding brain regions through an ephaptic coupling. These reasons aside, it is also of interest to examine whether our algorithm can stabilize ictal activity in non-localizing seizure samples. Similar to localizing samples, seizure onsets in non-localizing samples are marked by abnormal activity in one or many electrodes. In supplementary figure 11, we demonstrate that similar to localizing seizures (supplementary figure 2), the changes in the eigenvector structure of the systems estimated following the unequivocal onsets, capture the emergence of ictal activity in non-localizing seizures. In supplementary figure 12, we also provide an in *silico* example of stabilization of ictal activity in a non-localizing seizure sample. Our findings highlight the need for intervention strategies that are informed not by phenomenological models agnostic to the underlying drivers of neural activity, but instead by biophysical mechanisms of seizure initiation and evolution.

### 3.4. Generalized pole placement allows offline tuning

In the previous section, we demonstrated that our method allows us to dampen ictal oscillations using only a small set of sensing and stimulating electrodes. Nevertheless, the implemented algorithm in the previous section requires real-time identification of the system parameters using all the implanted electrodes. Here, we demonstrate that our proposed method in patients with stereotypic seizures may also allow offline estimation of the feedback gains.

**Figure 5.**
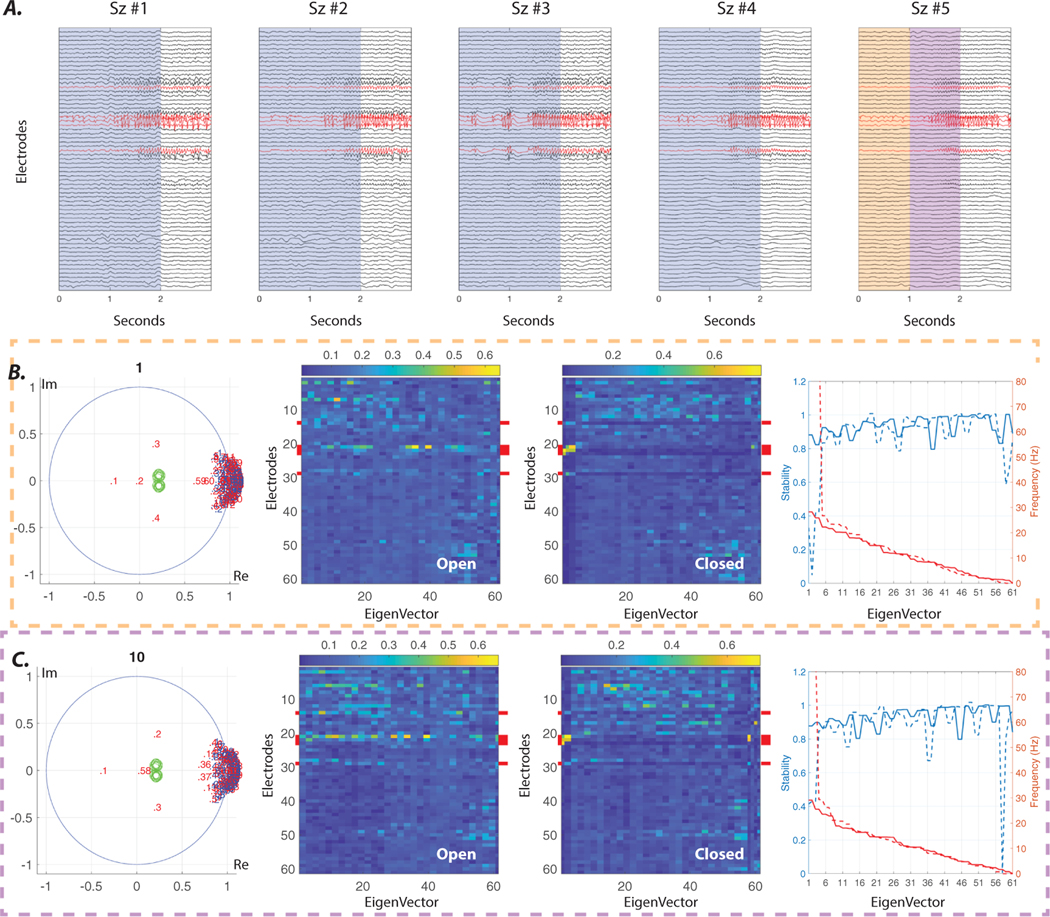
Offline estimation of closed-loop feedback gains. (A) Five examples of iEEG recordings at the time of seizure onset from a single patient with well-localized SOZ. (B) The left panel shows the distribution of the estimated eigenvalues at the onset of seizure #5 (orange window in panel A). Eigenvalues are sorted based on their frequencies, from highest to lowest. Blue numbered dots represent the empirically estimated values. We simulate the effect of closed-loop static feedback between a few electrodes (marked by red in panel A), by representing eigenvalues of the closed system (red numbered dots). Only five electrodes with the highest eigenvector loading at seizure onset (as seen in panel B) were selected as stimulating and sensing electrodes to mimic the limited channels of implantable neurostimulation devices. Similar to figure 4, the static output feedback gains were calculated using the generalized pole placement method [31], with the control-theoretic objective of shifting all the higher frequency (> 15 Hz) eigenvalues of the systems that were estimated from 40 sliding windows (4 seizures × 10 consecutive sliding windows) following the onsets of seizures #1–4 to the predefined zones represented by green circle (for more details see supplementary information). The two middle panels show the absolute values of eigenvectors of the open and closed systems estimated from seizure #5. The right panel shows the stability (i.e. absolute values of eigenvalues) and frequency of all eigenvalues in both open (solid lines) and closed (dashed lines) systems estimated from the same seizure. Note the reduced stability of all the closed system eigenvalues with associated eigenvectors with high loading on stimulating electrodes (marked by red in the middle panels), namely eigenmodes 1–4 (see supplementary figure 4 for statistical test results). (C) Same results as in panel B, calculated for the purple window in panel A. Broadly, this simulated example suggests that the static feedback gains calculated *offline* from patients with stereotypical seizures might be utilized to stabilize the seizure onset activity using only a few sensing and stimulating electrodes, without the need for any real-time analysis.

We consider five seizure samples from one patient (HUP70) with stereotyped seizures that have clear localization of the SOZ (figure 5(A)). In this example, we treat seizures #1–4 as our offline samples and calculate the feedback gains by considering all seizure onset sliding-windows (1 s window with 100 ms increments) across the four seizures (i.e. 4 × 10 = 40 windows). We show that these feedback gains can then be used to reduce the stability of the ictal oscillations in seizure #5 over time (figures 5(B)–(C)). This result is a proof of concept that it might be possible to leverage our method for offline tuning of the closed-loop feedback gains in patients with stereotyped seizures, and that the control objective can be achieved using only a few sensing and stimulating electrodes due to the focal nature of well-localized seizure onset zones.

## 4. Discussion

### 4.1. Dynamic stability analysis of seizure onset dynamics

To date, several computational methods have been proposed to study seizure dynamics and its spatiotemporal evolution as reflected in iEEG. Naturally, methods to examine the directed or effective connectivity [51] have been widely used to establish the influence that neural populations exert over each other around the onset of a seizure. Multivariate autoregressive (MVAR) modeling, Granger causality [52], partial directed coherence [53], and directed transfer [54] are related approaches to study effective connectivity in multichannel iEEG signals. These measures have been used to study the *flow* of seizures from one channel or source to another, as well as to identify SOZ electrodes [20, 55–60].

A longstanding theory regarding the dynamical progression of seizures is that they evolve through different stages or dynamical states [12, 37, 61, 62]. However, the aforementioned methods assume that the underlying system is stationary over the window of estimation. In order to address these limitations, adaptive versions of the methods above have been introduced, and have been shown to capture the time-varying nature of ictal onset dynamics [19, 25, 63–66]. For instance, Mullen, Worrell [16] demonstrated that principal oscillation patterns estimated from spatially fixed sources decomposed from the iEEG time series reveal distinct shifts in characteristic frequency and damping time of a small subset of the most dynamically important eigenmodes, time-locked to ictal onset. Together, these time-varying linear models allow us to capture the changes in the underlying system, and to characterize the ictal dynamics.

Here, towards our ultimate goal of model-based control of seizures, we considered the dynamics at the ictal onset to be characterized by emerging focal oscillations and we, therefore, used adaptive MVAR to identify the parameters of a linear model of seizure sources. We first provided a synthetic example of time-varying oscillations to build intuition about how the emergence of focal sources can be captured via dynamical stability analysis of the identified model of the system. Our simulations reveal that spatiotemporally overlapping and time-varying oscillations are marked by fluctuations in the frequency and damping profile of the identified systems and that dynamical stability analysis accurately uncovers the planted oscillations following these periods of transition. Next, we examined seizure onset dynamics in patients with clear localization of the SOZ on the iEEG and show that in line with prior reports (e.g. [16, 22, 58, 59]), adaptive MVAR reliably highlights the focal ictal sources at the SOZ. We demonstrate that an increase in the kurtosis and maximum value of eigenvectors captures the sudden changes in the spatial profile of the eigenvectors of the identified model of the system at seizure onset. Together, these observations highlight the utility of linear models of seizure dynamics, which we then use to inform a proposed seizure intervention strategy.

Our in-depth analysis of the SOZ through the lens of an LTI model was performed with the goal of providing an accurate characterization of seizure onset activity, which we then use to inform a proposed seizure intervention strategy. Further, we do not claim to offer a novel methodology for SOZ localization, but rather we build upon the insights previously obtained from dynamical systems modeling and stability analysis.

### 4.2. Implantable neurostimulation devices and model-based design for seizure control

Decades of research have demonstrated that field effects can modulate the threshold of neuron excitability [67–71]. Field effects induced by endogenous brain activity can also causally affect neural dynamics via ephaptic coupling [72]. Moreover, field effects are believed to play an important role in the initiation and propagation of seizures [73, 74]. For instance, evidence suggests that field effects are likely sufficient to generate and sustain the high synchronization observed at the ictal core [72, 75–78] (for a recent review see [30]). Moreover, *in vivo* laboratory experiments have shown that electrical stimulation can alter the LFPs and consequently modulate the firing of neurons [68, 79–84]. Motivated by these observations, many studies have experimented with injecting current into the seizure onset zone aimed at suppression or control of ictal activity [85–91].

Closed-loop feedback provides a simple yet practical framework for seizure intervention, and to date, several *in vivo*, as well as modeling, studies have provided evidence of their potential for control of ictal activity [48, 83, 87–89]. For instance, Gluckman and colleagues demonstrated that providing local negative feedback can ameliorate seizure-like activity in hippocampal brain slices. Here, we also consider closed-loop feedback control, but unlike the studies above, the feedback gains are calculated based on a *model* of the system. More speculatively, the features that we obtain from the eigenmode decomposition could be used as quantifiable performance objectives for optimizing stimulation parameters in closed-loop implantable devices [3, 92]. Specifically, this approach would depart from the current practice of manual, intermittent, off-line tuning of deep brain stimulation parameters such as amplitude and frequency based on the count of seizures experienced by the patient. Instead, the features estimated from the dynamical stability-based approach that characterize seizure onset can be used to select channels for feature extraction in a closed-loop assessment and stimulation paradigm [93].

We proposed spectral control of cortical activity as an early seizure intervention strategy in our prior work [31]. Based on prior reports of the excursions of frequency and stability of the oscillatory eigenmodes [16], we proposed a control objective that aims to maintain the spectral profile of the system following the seizure onset [31]. However, by examining the eigenvectors of the estimated system around the seizure onset, here we demonstrate that the fluctuations in frequency and stability mark a transition the emergence of focal ictal sources. Since our generalized pole placement scheme [31] is agnostic to the changes in the spatial profile of the system, we proposed an alternative control strategy that aims to damp the focal ictal seizure sources. Moreover, we provide simulations and show that static output feedback, in theory, can successfully dampen focal seizure activity. Although the computational burden exceeds the capability of currently implantable platforms, recent work by our group indicates that there is marked potential in hybrid implantable/cloud-based platforms for data reduction and off-device computation for analysis and control [94].

Here we provided a tailored design for patients commonly considered ideal candidates for treatment with neurostimulation. Nevertheless, our approach could have potential impact on patients with multiple or unclear onset zones. Although the current generations of neurostimulators have limited onboard computational power that may be restrictive for real-time control of seizures with multiple or changing foci, our framework is not limited in theory and in fact is also suitable for automated control of pathological oscillations of the electrical field in seizures with changing morphology and locus.

Nevertheless, our synthetic example demonstrates that our theory and proposed algorithm also allows offline determination of feedback gains, which could potentially be estimated in patients with stereotypical seizures using only a small set of electrodes. Based on our results, we also conjecture that the placement of the few electrodes afforded by neurostimulation devices can be guided based on the location of the electrodes for which eigenvalue-eigenvector features provide reliable estimates of focal seizure sources in pre-surgical iEEG recordings. Taken together, our numerical experiments highlight the theoretical utility of the model-based approach towards seizure control via neurostimulation.

Our work is also coherent with recent computational network models of epilepsy such as the fragility- and synchronizability-based approaches [95, 96], although these models have different assumptions and utility. The fragility framework uses similar principles as the method adopted in our work in several ways. Specifically, both methods use LTI models and target the stability of the modeled systems. However, similar to the synchronizability, the fragility approach is primarily a resective framework. Fragility allows the investigator to identify brain regions whose removal results in the maximal reduction in the stability of the remaining network. The authors argue that this reduced stability, in theory, decreases the pro-ictal propensity of the network. In contrast, the synchronizability framework provides insight into how the topology of networks of coupled oscillators allows for sustained synchronous ictal activity. Therefore, similar to the fragility approach, it is a fruitful avenue for understanding how cortical resections and lesions change the network topology, which in turn will affect the global pathological dynamics. Although the intervention strategies between our method and synchronizability are quite different, both methods can be considered in a unified view. The synchronizability framework can, in theory, explain the emergent macroscale behavior of networks of neural populations with nonlinear oscillatory behavior, while linearization and state feedback control can describe and regulate the dynamics of this nonlinear system locally around an operating point and offer an objective for real-time control via neurostimulation.

### 4.3. Methodological considerations

Several methodological considerations are pertinent to this work. The proposed analysis does not directly allow for the assessment of underlying biological mechanisms. Although it is likely that the underlying dynamical process captured by iEEG data is nonlinear, the dynamical *stability*-based approach allows for the analysis of global non-stationarity and nonlinear dynamics by fitting a linear model to short time windows. Moreover, prior work has demonstrated that iEEG recordings can be leveraged for feedback control of seizures with relative success in animal models of epilepsy (e.g. [83]). Nevertheless, future research could examine the limitations of our adopted class of phenomenological models in experimental and *in vivo* setting, and establish the robustness and dependence of prior reports on the state of the underlying system. For example, following the seizure onset, the ictal core regime may provide a better opportunity for feedback control of neural populations using direct electrical stimulation, because the activity of the neural population at the ictal core is strongly governed by field effects(for review see[30]).

In this work, the choice of number and location of sensing and stimulating electrodes were clinically motivated and were informed by the limitations of current neurostimulation devices. Nevertheless, future work should examine the dependence of controllability and observably of the estimated system and our ability to regulate its dynamics on these parameters. The results from these studies can offer an avenue for model-based electrode selection aimed at closed-loop control of seizures.

Moreover, in our proof-of-concept examples, we strategized to stabilize a rather arbitrary range of high-frequency oscillations. Although our algorithm is agnostic to these choices, in practice the response of cortical tissue to direct electrical stimulation is selective [97].Future work could inform our proposed control strategy experimentally by identifying which frequencies provide better targets for intervention. In addition, some degree of offline tuning of parameters is required for the calculation of the feedback gains, but we showed that it could be trained offline for patients with stereotypical seizures.

Importantly, our numerical experiments assume that direct stimulation does not change the dynamical properties of the underlying seizure source. However, in practice, the underlying system may change following direct electrical stimulation. We optimistically conjecture that this change may come about from the disrupted synchronization of the neural population at the ictal core due to the targeted damping of the oscillations of the local electric field. Alternatively, electrical stimulation can also cause changes in the dynamical properties of the underlying system without seizure termination, which necessitates real-time re-identification of system parameters and feedback gains.

Adaptive linear control is based on an approximation of a linear function that is accurate only in a small neighborhood around an operating point. In theory, one can expand the neighborhood where the approximation is accurate using nonlinear models. Nevertheless, the nonlinear controller also may not achieve regulation objectives, if the trajectories of the states move beyond the approximation region. Moreover, our results demonstrate that the estimated first-order system depends on spatiotemporal sampling and is likely to contain approximation error. Although we can only speculate on the effects of the these factors on our ability to control ictal oscillations in practice, future work can aim to address some of these limitations, such as improving the goodness-of-fit of the LTI model using Kalman filtering [98].

Here we demonstrated that the number of stimulating and sensing electrodes limits the number of eigenvalues that are successfully placed close to the zone with low stability. Nevertheless, our observations suggest that due to the focal nature of seizures, the ictal states are observable with only a few electrodes. These results suggest that implantable devices with only a few electrodes, placed at strategic locations can, in theory, allow effective control. We identified stimulating electrodes based on the loading of electrodes in eigenvectors associated with the emerging high-frequency ictal oscillations. Future work could aim to improve the efficacy of our intervention strategy by informing the locus of the electrodes based on the observability of the targeted ictal state.

While the goal of our work is not seizure detection, our dynamical stability-based approach allows us to characterize the changes in the underlying system following the seizure onset. That said, the normal (non-epileptic) ongoing brain activity also shows a rich repertoire of multi-scale oscillatory transients. Therefore, further efforts in seizure detection and control could benefit from an examination of ictal as well as inter-ictal oscillations across spatiotemporal scales. In the context of control, the integration of that additional information into the neurostimulation devices may be limited by considerations of energy consumption, the number of electrodes, and computational power afforded by the current devices.

Future validation experiments should bear in mind that here the closed-loop control is performed only on the linearization of the cortical dynamics. Additional nonlinear effects could be captured by considering feedback linearization, which would reduce the problem to the same setup presented in this paper. In practice, however, it is likely that unexpected behaviors will arise when applying these strategies in a clinical setting, and some additional nonlinear modeling may need to be considered.

Finally, we have demonstrated that the dynamical stability analysis allows us to identify similar changes in the underlying process during the seizure onset period for different samples across patients. Despite our rather homogeneous dataset, which includes a large number of seizures with overlapping features, heterogeneity is still present in the evolution of seizures across subjects and samples. Our clinical assessment reveals that the major source contributing to this heterogeneity, which also has critical implications for model-based intervention strategies, is the localization quality of the epileptic network and seizure onset zone. Our clinical assessments reveal that only a handfull ofdatasets can be labeled as localizing with a high degree of confidence. These observations highlight the crucial need for the development of machine learning algorithms capable of accurate pre-surgical assessment of the quality of localization of epileptic networks. Many other factors such as the location and number of electrodes, sampling rate, recording noise, and type of seizures may also partially explain the subtle observed inter-subject variations. Nevertheless, the dynamical system analysis provides an avenue for the characterization of idiosyncratic features can be leveraged for targeted model-based control.

### 4.4. Future directions

The first few studies validating the use of this approach for closed-loop control could be retrospective. Epilepsy Centers could pool data from the ~ 1000 patients implanted with NeuroPace RNS devices and compare successful stimulations to unsuccessful stimulations. New studies of next-generation responsive stimulation devices from several companies are underway and these stimulation and modeling paradigms could be performed in animal models of epilepsy, perhaps starting with epileptic canines, to test these hypotheses [99]. These strategies could also be tested in patients admitted for epilepsy monitoring with intracranial electrodes during functional stimulation, to arrest and control ictal events that routinely arise during brain mapping with electrical stimulation (not intentionally provoked), similar to the first NeuroPace studies [10]. In performing these studies, one would need to consider computational complexity and interfacing device hardware with algorithm software, although this is routinely done with bedside computers splitting off the iEEG signal.

For outpatient implementations, the required computational power is another important consideration. Our group is currently coupling implantable devices to cloud-based computation to augment implanted devices, either directly or through handhelds that communicate with the device and cloud-based platforms, such as Blackfynn. Intelligent, closed-loop stimulation has great promise to help patients with neurostimulation and epilepsy, and the options for tracking and responding to complex iEEG patterns in patients are increasing. Success with empirically derived stimulation paradigms is already positively impacting many patients with refractory, inoperable epilepsy, although results are still modest. We hope that applying new control strategies to these devices, as engineers have done for many other non-biological systems, will greatly augment the efficacy of current and new implantable anti-seizure devices.

## 5. Summary

We provide a dynamical stability-based characterization of the onset of seizure activity in well-localized epileptic network that hinges on an eigenmode decomposition of a computationally tractable model. We demonstrate an approach that uses eigendecomposition of the estimated model parameters to reveal the stereotyped behavior of well-localized epileptic networks at the onset of seizures, which can be leveraged to classify, identify, and potentially control seizures. We propose a novel control strategy that aims to stabilize oscillatory modes of the estimated system following seizure onset by providing static-output feedback. Using system parameters estimated from a sample seizure, we demonstrate that the feedback gains afforded by a generalized pole placement method offer potential solutions for stabilizing seizure onset dynamics and can potentially allow offline tuning of neurostimulation parameters. Together, these results demonstrate that our model-based approach and proposed control strategy, in theory, could inform the stimulation strategies of implantable devices and improve their efficacy.

## Supplementary Material

supplementary

## Figures and Tables

**Table 1.** Patient Information. The subjects from the University of Pennsylvania and Mayo Clinic (Rochester) cohorts were labeled as HUP and study, respectively. For each patient, we report sex, as well as age at first reported seizure onset and at phase II monitoring (age). Additionally, we report the seizure etiology, which was clinically determined through medical history, imaging, and long-term invasive monitoring. The different seizures observed (seizure types) include simple-partial (SP), complex-partial (CP), and complex-partial with secondary generalization (CP + GTC). We also indicate the total number of seizures recorded in the epilepsy monitoring unit, as well as the clinical imaging analysis (imaging) that concludes whether the seizure etiology is lesional (L) or non-lesional (NL). Finally, surgical outcome (outcome) was based on either Engel score or ILAE score: seizure freedom to no improvement (I–V), no resection (NR), and no follow-up (NF). Finally, patients are labeled as localizing (L) or non-localizing (NL) based on the localization quality of the seizure onset zone (SOZ localization) after clinical assessment of the iEEG by a board-certified epileptologist (F.M.).

Patient (IEEG Portal)	sex	Age(Years) (Onset/Surgery)	SeizureOnset	Etiology	Seizure Type	Seizures (N)	Imaging	Outcome	SOZ Localization
HUP64_phaseII	M	0.3/20	Left frontal	Dysplasia	CP + GTC	01	L	ENGEL-I	L
HUP65_phaseII	M	02/36	Right temporal	Dysplasia	CP + GTC	03	N/A	ENGEL-I	NL
HUP68_phaseII	F	15/26	Right temporal	Meningitis	CP, CP + GTC	05	NL	ENGEL-I	L
HUP70_phaseII	M	10/32	Left perirolandic	Cryptogenic	SP	08	L	NR	L
HUP72_phaseII	F	11/27	Bilateral left	Mesial temporal sclerosis	CP + GTC	01	L	NR	L
HUP73_phaseII	M	11/39	Anterior right frontal	Meningitis	CP	05	NL	ENGEL-I	NL
HUP78_phaseII	M	00/54	Anterior left temporal	Traumatic injury	CP	05	L	ENGEL- III	L
HUP79_phaseII	F	11/39	Occipital	Meningitis	CP	01	L	NR	NL
HUP86_phaseII	F	18/25	Left temporal	Cryptogenic	CP + GTC	02	NL	ENGEL- II	L
HUP87_phaseII	M	21/24	Frontal	Meningitis	CP	02	L	ENGEL-I	NL
Study 004–2	F	14/27	Right temporal occipital	Unknown	CP + GTC	01	NL	ILAE-IV	NL
Study 006	M	22/25	Left frontal	Unknown	CP	02	NL	NR	NL
Study 010	F	00/13	Left frontal	Neonatal injury	CP + GTC	02	L	NF	L
Study 011	F	10/34	Right Mesial Frontal	Unknown	CP	02	NL	NF	L
Study 016	F	05/36	Right temporal orbitofrontal	Unknown	CP + GTC	03	NL	ILAE-IV	L
Study 019	F	31/33	Left frontal	Unknown	CP + GTC	15	NL	ILAE-V	NL
Study 020	M	05/10	Right frontal	Unknown	CP + GTC	04	NL	ILAE-IV	L
Study 023	M	01/16	Left occipital	Unknown	CP	04	L	ILAE-I	NL
Study 026	M	09/09	Left frontal	Unknown	CP	10	NL	ILAE-I	NL
Study 031	M	05/05	Right frontal	Unknown	CP + GTC	05	NL	NF	NL
Study 033	M	00/03	Left frontal	Unknown	GA	07	L	ILAE-V	NL
Study 037	F	45/??	Indeterminate	Unknown	CP	02	NL	NR	NL
