# Supplementary material for: Model-based design for seizure control by stimulation
Source: J Neural Eng. Author manuscript; Available in PMC 2021 Aug 5. (PMC8341467; doi:10.1088/1741-2552/ab7a4e)
Supplement: supplementary [file NIHMS1703860-supplement-supplementary.pdf]

Supplementary Materials

for

"Model-based design for seizure control by stimulation"

Non-localizing iEEG samples

iEEG electrodes are commonly implanted in select patients undergoing presurgical assessment, where non-invasive neuroimaging methods failed to provide accurate localization of the epileptic network and the seizure onset zone. Although a wide array of rigorous clinical (e.g., seizure semiology) and neuroimaging (e.g., structural MRI and PET scans) assessments are used to guide the placement of iEEG electrodes, some cases remain in which the electrodes still fail to accurately localize the epileptic networks. Since the successful localization of the seizure onset zone and the epileptic network via iEEG is pertinent to our ability to model and ultimately control the underlying seizure dynamics, here we only focus only on seizure samples from patients labeled as "localizing" through clinical assessment (F.M.). Non-localizing iEEG datasets commonly exhibit patterns of seizure propagation marked by the presence of diffuse and slow seizure onset activity. The absence of the LVFA and sharp rhythmic spiking was the most important feature of the iEEG datasets with poor localization of the SOZ (see SI Figure 1). We provide a list of "localizing" patients, or those with a clear localization of the SOZ, in Table 1.

Changes in the distribution of eigenvectors following seizure onset

Focal seizures in the localizing iEEG datasets are marked by the emergence of epileptiform activity from the electrodes located in the vicinity of the seizure onset zone. As demonstrated in Figure 2 of the main text, eigenvectors of the system estimated in the peri-ictal regime can reflect these emerging focal oscillations. The changes in the maximum value and kurtosis of the eigenvectors following the seizure onset effectively highlight the shift from the disordered preictal regime to the highly ordered and focal ictal onset regime. We used these measures to demonstrate the similarity between focal seizure samples in our dataset.

Here we demonstrate that the seizure onsets are marked by a strong increase in the kurtosis and maximum value of the estimated eigenvectors associated with oscillations across a wide range of frequencies (see SI Figure 2). We tested the statistical significance of these observations by comparing the pre- and post-seizure onset distributions of the normalized kurtosis and maximum value of the eigenvectors estimated from all iEEG samples with well-localized seizure onset zones. As seen in SI-Figure 2, the normalized kurtosis and maximum values are significantly lower (Wilcoxon rank-sum test, $p < 0.05$) in a 5 sec preictal window compared to values calculated from a window with the same length immediately after both the earliest electrographic and unequivocal electrographic onsets across several frequency bands. These results indicate that the emergence of ictal sources is similarly reflected in the eigenvectors of the system, as focal oscillatory eigenmodes across a wide range frequencies.

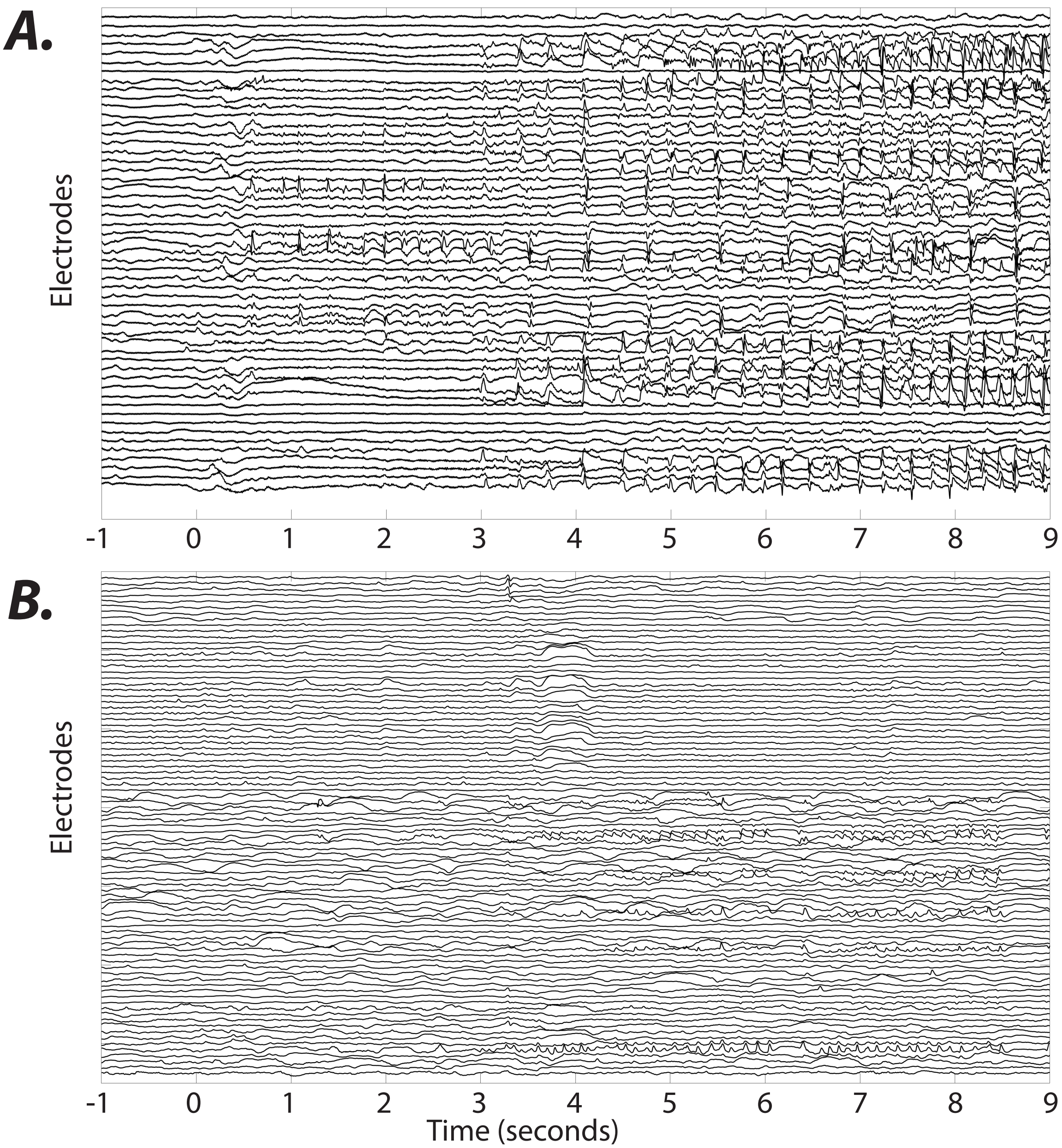

Figure 1: Examples of non-localizing iEEG recordings. Here we provide two example seizures from two patients with non-localizing iEEG recordings, namely Study 004-2 **(A)** and Study 019 **(B)**. Note that the seizure onsets (t=0) are marked by the emergence of diffuse and slow oscillations across several electrodes.

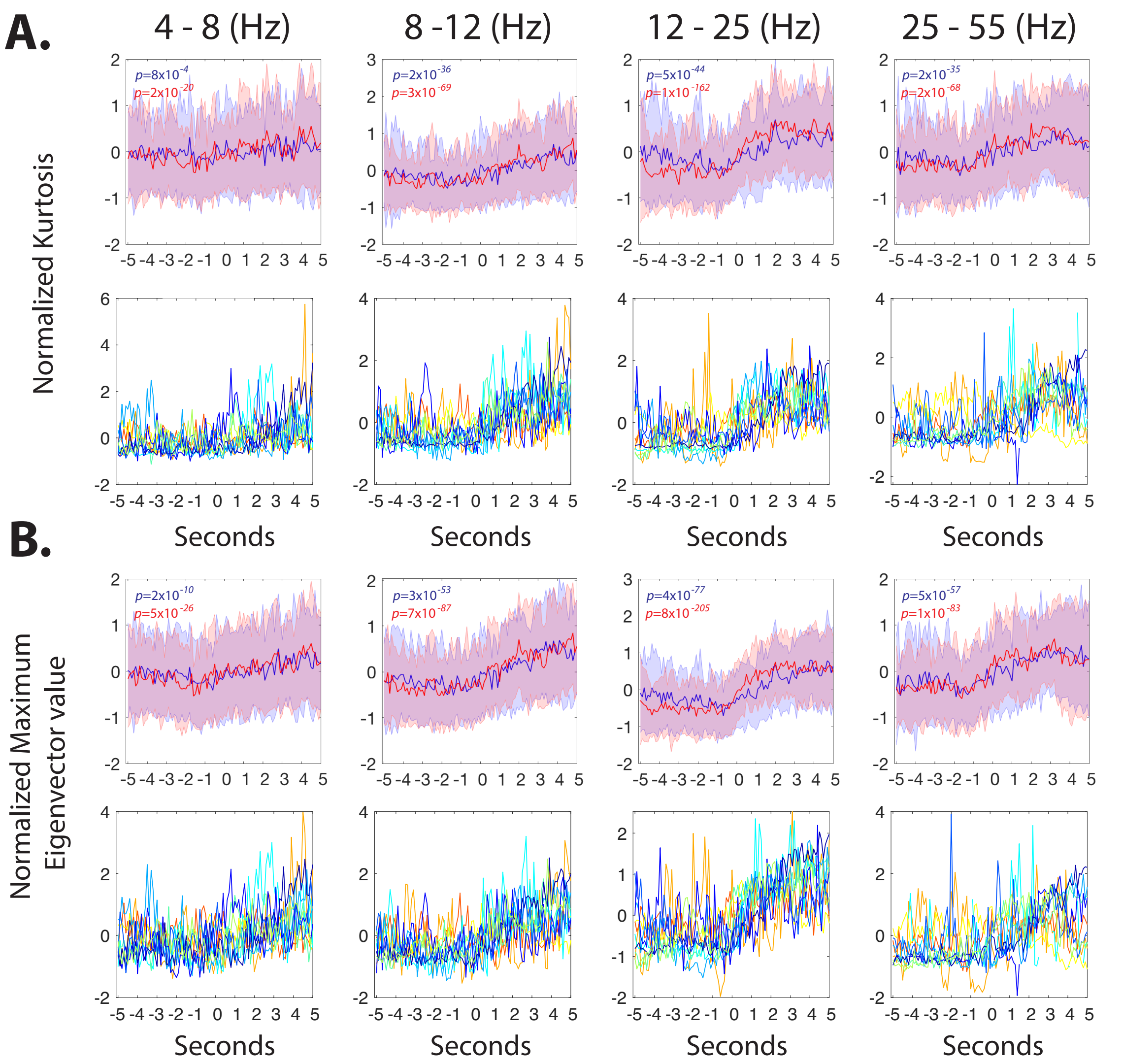

Figure 2: The wide-band increase in the maximum value and kurtosis of the estimated eigenvectors following seizure onset. (A) Top panels show the average normalized kurtosis calculated from eigenvectors associated with different frequency bands at the earliest electrographic change (blue line) and at the unequivocal seizure onset (red line) across all localizing samples. The x-axis represents the time from seizure onsets. The shaded area represents the standard deviation of the samples. A non-parametric Wilcoxon rank-sum test reveals that seizure onset is marked by a wide-band significant increase ($p < 0.05$) in the normalized kurtosis values concatenated across all seizure samples over a 10-second peri-ictal time window. The color-coded curves in the bottom panels show the average normalized kurtosis calculated for each patient separately following the unequivocal seizure onset. **(B)** The top panels show the average normalized maximum values calculated from eigenvectors associated with different frequency bands at the earliest electrographic change (blue line) and at the unequivocal seizure onset (red line) across all localizing samples. The shaded area represents the standard deviation of the samples. Similar to panel A, we used a non-parametric Wilcoxon rank-sum test and observed that the seizure onset is marked by a wide-band significant increase ($p < 0.05$) in the normalized kurtosis values concatenated across all seizure samples over a 10-second peri-ictal time window. The color-coded curves in the bottom panels show the average normalized kurtosis calculated for each patient separately following the unequivocal seizure onsets.

Generalized pole placement method

The effect of direct electrical stimulation using the linear model can be captured by the following equation

$$x_{k+1} = A_k x_k + B u_k + \epsilon_k, k = 0,1, \dots, \quad (1)$$

where $u_k \in \mathbb{R}^p$ is the input injected at time k. The states of the sensors, i.e., the state of the system, can be also identified from the measured output of the system, i.e., cortical activity, and can be described as

$$y_k = C x_k \,, \quad (2)$$

where $y_k \in \mathbb{R}^m$correspond to the data collected at time k by the intracranial electrodes. We propose using a static output feedback controller of the form

$$u_k = K y_k, \quad (3)$$

and then the closed-loop dynamics is given by

$$x_{k+1} = (A_k + BKC) x_k + \epsilon_k. \quad (4)$$

As a seizure intervention control strategy, we propose a closed-loop actuation, with the aim of driving the eigenvalues of the system closer to the center, towards the asymptotically stable zone and, thus, in effect damping the focal ictal onset dynamics. This problem can be studied as a particular case of the *generalized switching pole placement problem* studied in our prior work [1] and formulated as follows.

Problem $\mathcal{P}_1$ Given a time-varying system (1) and (2), static output feedback described by (3), and a collection of closed subsets $\mathcal{C}_1, \dots, \mathcal{C}_n \subset \mathbb{C}$ where we want the eigenvalues of the closed-loop system to be contained, determine K such that

$$\lambda_i^k(A_k + BKC) \in \mathcal{C}_i^k, i = 1, \dots, nk = 0, \dots$$

where $\lambda_i^k(A_k + BKC)$ is the i-th eigenvalue of the closed-loop system described in (4).

Here, we introduce the terminology and basic notions used in the rest of this section for readers. For a given matrix $M \in \mathbb{C}^{r\times s}$, the vectorization operator $\mathrm{vec}(M) \in \mathbb{C}^{rs}$ consists of the columns of M stacked below each other. We denote by $\mathbf{0}_{r\times s}$ the zero matrix in $\mathbb{C}^{r\times s}$, and, given matrices $Y \in \mathbb{C}^{m\times n}$ and $Z \in \mathbb{C}^{p\times q}$, the Kronecker product between $Y$and Z denoted by the $mp\times nq$ matrix $Y \otimes Z$. Finally, we denote by $\mathrm{Re}(Z) \in \mathbb{R}^{r\times s}$ and $\mathrm{Im}(Z) \in \mathbb{R}^{r\times s}$ the real and imaginary parts of a matrix $Z \in \mathbb{C}^{r\times s}$.

Now, let x be an element and $\mathcal{C}$ be a closed (not necessarily convex) set in a Hilbert space $\mathcal{H}$. Any $c_0 \in \mathcal{C}$ such that $\| x - c_0 \| \leq \| x - c \|$ for all $c \in \mathcal{C}$ is referred to as a *projection of* x *onto* $\mathcal{C}$. In our case, we deal with finite-dimensional Hilbert spaces, where there is always at least one such point. It is known that if $\mathcal{C}$ is a closed convex set then each point in $\mathcal{H}$ has only one projection onto $\mathcal{C}$. Then, one can introduce the *projection operator* onto $\mathcal{C}$ that is a function $\mathcal{P}_{\mathcal{C}}: \mathcal{H} \to \mathcal{H}$ such that for each $x \in \mathcal{H}$ it returns the projection of x onto $\mathcal{C}$, i.e., $\mathcal{P}_{\mathcal{C}}(x)$.

We focus on a particular case of the following problem: given closed sets $\mathcal{C}_1, \dots, \mathcal{C}_N$ in a finite-dimensional Hilbert space $\mathcal{H}$, determine a point in the intersection $\bigcap_{i=1}^{N} \mathcal{C}_i$ (assuming this to be non-empty. Assuming all the sets are closed and convex, then this

problem is solvable using the alternating projections method [2]. If the sets are non-convex, then no global convergence guarantees exist, and also for different initial conditions the alternating projections method may not converge [3].

In our previous work [1], we show that solving problem $\mathcal{P}_1$ is computationally hard (i.e., NP-hard) and consequently, it is unlikely that an algorithm exists that solves $\mathcal{P}_1$ polynomially. Therefore in problem $\mathcal{P}'_1$, we propose a reformulation of $\mathcal{P}_1$ as the problem of determining the gain K that lies in the intersection of several sets.

Let $\mathcal{L}_k$ be the set of all possible closed-loop matrices for the different evolution matrices A_k, described by

$$\mathcal{L}_k = \{L \in \mathbb{R}^{n\times n}: L = A_k + BKC \text{ for some } K\},$$

where $k = 1, \dots, T$, and T a finite-time horizon, and let $\mathcal{M}_k$ be the set of complex matrices with eigenvalues in the specified regions $\mathcal{C}_1^k, \dots, \mathcal{C}_n^k$ given by

$$\mathcal{M}_k = \{Z \in \mathbb{C}^{n\times n}: \lambda_i(Z) \in \mathcal{C}_i^k, i = 1, \dots, n\}$$

for $k = 1, \dots, T$. Therefore, problem $\mathcal{P}_1$ can be solved by addressing the following problem.

Problem $\mathcal{P}'_1$ find $X \in \mathcal{L}_1 \cap \dots \cap \mathcal{L}_T \cap \mathcal{M}_1 \cap \dots \cap \mathcal{M}_T$ ∘

Therefore, K that solves problem $\mathcal{P}_1$ (or, equivalently, problem $\mathcal{P}'_1$) can be obtained as the minimizer of $\| (C^\top \otimes B)\text{vec}(K) - \text{vec}(\text{Re}(X) - A_1) \|_2$, since K lies in the intersection of the sets $\mathcal{L}_k$ that are convex sets, whose projection can be computed as follows.

Lemma 1 ([4]): The projection of $X \in \mathbb{C}^{n\times n}$ onto $\mathcal{L}_k$ is given by $\mathcal{P}_{\mathcal{L}_k}(X) = A_k + BKC$ where $K \in \mathbb{R}^{p\times m}$ is a minimizer of $\| (C^\top \otimes B)\text{vec}(K) - \text{vec}(\text{Re}(X) - A_k) \|_2$.

Nevertheless, $\mathcal{M}_k$ *is non-convex, and no easily computable projection scheme is available. Therefore, instead, we propose to use the following approximation* $\tilde{\mathcal{P}}_{\mathcal{M}_k(X)}$.

Definition 1 ([4]): Let $\mathrm{X} = \mathrm{VTV}^*$ be the Schur's decomposition, where $\mathrm{V} \in \mathbb{C}^{\mathrm{n}\times\mathrm{n}}$ is a unitary matrix and $\mathrm{T} \in \mathbb{C}^{\mathrm{n}\times\mathrm{n}}$ an upper triangular matrix. The approximate projection mapping $\tilde{\mathcal{P}}_{\mathcal{M}_k}(X)$ of X onto $\mathcal{M}_k$ is given by $\tilde{\mathcal{P}}_{\mathcal{M}_k}(X) = V\hat{T}V^*$, where

$$\hat{T}_{ij} = \begin{cases} \mathcal{P}_{\mathcal{C}^k_{\sigma^*(i)}}(T_{ii}) & \text{if } i = j, \\ T_{ij} & \text{otherwise}, \end{cases}$$

and $\sigma^* \in \Sigma$, with Σ denoting the set of possible permutations, minimizes the following optimization function. ◊

The mapping in Definition (1) is motivated by the fact that if X is a symmetric matrix, then $\tilde{\mathcal{P}}_{\mathcal{M}_k}(X)$ is the best approximation of X in the Frobenius norm *[4]*. Further, the permutation σ^* in Definition (1) can be determined by reducing the problem to a minimum weight maximum matching. This reduction process consists in determining permutation matrices M_1 and M_2 such that they minimize the trace$(M_1 \Theta M_2)$ where Θ is

the matrix whose entry (i, j) contains the value $|T_{ii} - \mathcal{P}_{\mathcal{C}_j^k}(T_{ii})|^2$, and $\mathcal{P}_{\mathcal{C}_j^k}(T_{ii})$ is the projection of the complex number T_{ii} onto the closed convex set $\mathcal{C}_j^k$. Therefore, σ^* can be described by the pairs $(i, j = \sigma^*(i))$ corresponding to the diagonal entries of $M_1 \Theta M_2$.

Next, in order to address $\mathcal{P}'_1$, in our previous work [1] we propose two variations of the alternating projections method, which involves sequentially computing the projection of a matrix in one of the sets, followed by finding the projection of the former in another set and so forth. Nonetheless, because of the projection on the sets $\mathcal{M}_k$ is only approximated by $\tilde{\mathcal{P}}_{\mathcal{M}_k}$, we propose to weight the *quality* of the projections in the alternating projection method. This quality is captured by a convex combination of the projection onto a convex set and the one onto a non-convex set. More specifically, we bias the approximation towards the projection onto the convex sets, whose projection can be exactly determined in a computationally efficient manner. For more details regarding the proposed algorithms see our previous publication [1].

Closed-loop feedback control using pole placement method

Seizure onset is marked by a transition from the disordered pre-ictal regime to the ordered regime following the ictal onset, marked by prolonged synchronized oscillations between several electrodes [5, 6]. Although the ictal-onset oscillations persist for several seconds, system parameters estimated using a sliding-window fluctuate. Here we demonstrate that the calculated feedback gains for stabilizing the estimated system's eigenmodes (see manuscript Figure 3 for details) using the pole placement method [7] only guarantee results for a single window. SI-Figure 3 demonstrates that the eigenmodes of the *closed* system associated with seizure onset oscillations are unstable ($|\lambda|>1$), even after one second, when we use the feedback gains calculated from the seizure onset window (SI-Figure 3.B). Together, these results show the high sensitivity of the outcomes to small fluctuations in the estimated system parameters, when the feedback grains are calculated using pole placement method from a single window.

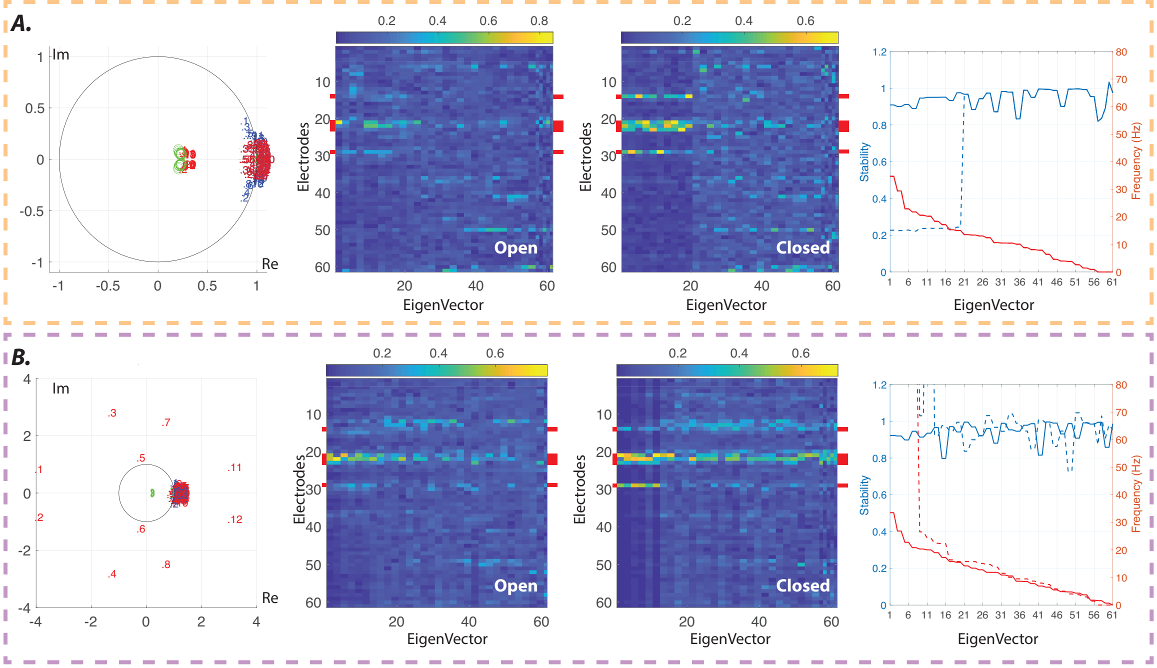

Figure 3: Pole placement closed-loop feedback control fails to stabilize ictal onset oscillations. (A) Similar to Figure 4.D in the main manuscript, here the left panel shows the distribution of the estimated eigenvalues at the seizure onset (orange window in panel A). Eigenvalues are sorted based on their frequencies, from highest to lowest. Blue numbered dots represent the empirically estimated values. We simulate the effect of closed-loop static feedback between the few chosen electrodes (marked by red in panel A), by representing the eigenvalues of the closed system (red numbered dots). The same five electrodes selected in Figure 4 of the main manuscript with the highest eigenvector loading at seizure onset (as seen in panel B) were selected as stimulating electrodes to mimic the limited channels of implantable neurostimulation devices. Nevertheless, unlike our proposed method, the pole placement method [7] requires all of the electrodes to also act as sensing electrodes. The static output feedback gains were calculated using the pole placement method with the control-theoretic objective of shifting all the higher frequency (>15 Hz) eigenvalues of the systems that were estimated from ten consecutive sliding widows following the seizure onset to the predefined zones represented by the green circle. Two middle panels show the absolute values of eigenvectors of the open and closed systems. The right panel shows the stability (i.e., absolute values of eigenvalues) and frequency of all eigenvalues in both the open (solid lines) and closed (dashed lines) systems. Note that the algorithm places all the selected eigenvalues at the predefined location with high accuracy. **(B)** Similar results as in panel A, calculated for the purple window in Figure 2.A in the main manuscript. These results demonstrate that the gains calculated from the previous 1-second window (orange window in Figure 2.A in the main manuscript) fail to stabilize the seizure onset dynamics and instead give rise to unstable oscillations, indicated by the presence of eigenvalues with stability values higher than 1 (See SI-Figure 4 for statistical test results)..

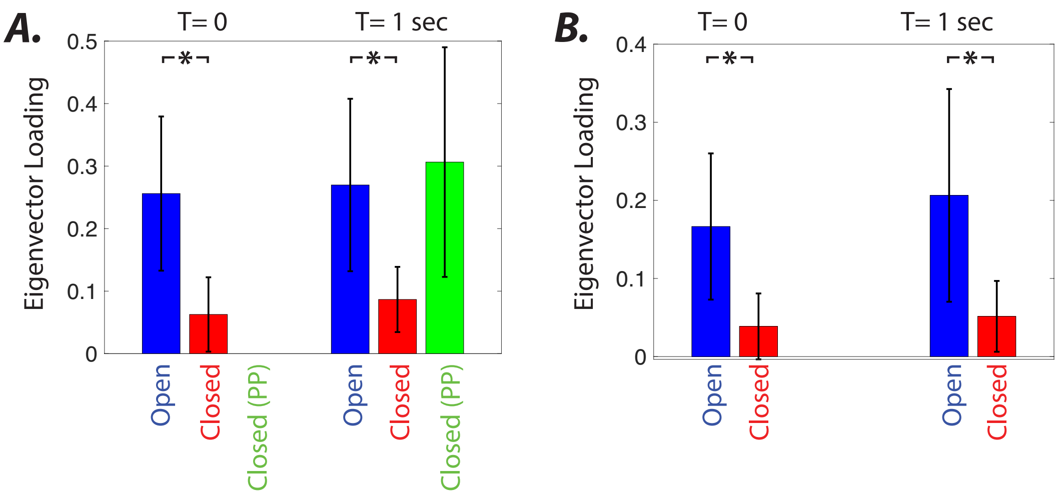

Figure 4: SOZ electrodes' eigenvector loading following the ictal onset for open and closed systems. **(A)** The blue bar represents the average loading of SOZ electrodes across higher frequency eigenvectors (>15 Hz) with high stability values (>0.6) for two time points, the seizure onset and the following one-second window for seizure sample in Figure 4 in the main text. The error bars represent the standard deviations around means. The red bars show the same values calculated from the closed system in Figure 4 in the main text. Non-parametric statistical testing (Wilcoxon rank-sum test, $p < 0.05$) shows that the loading of SOZ channels is significantly reduced at both time points. The green bar shows the same values calculated from the closed system in SI-Figure 3 using the pole placement (PP) method. Note that all high-frequency eigenmodes have low stability values, which explains the absence of the green bar at $t = 0$. However, the loading grows at the next one-second window and the closed system becomes unstable (as seen in SI-Figure 3). **(B)** Similar to panel A, bar plots represent the average loading of SOZ electrodes across higher frequency eigenvectors (>15 Hz) with high stability values (>0.6) for the seizure sample in Figure 5. Note that similar to panel A, the eigenvector loadings reduce significantly (Wilcoxon rank-sum test, $p < 0.05$) at both time points in the closed system.

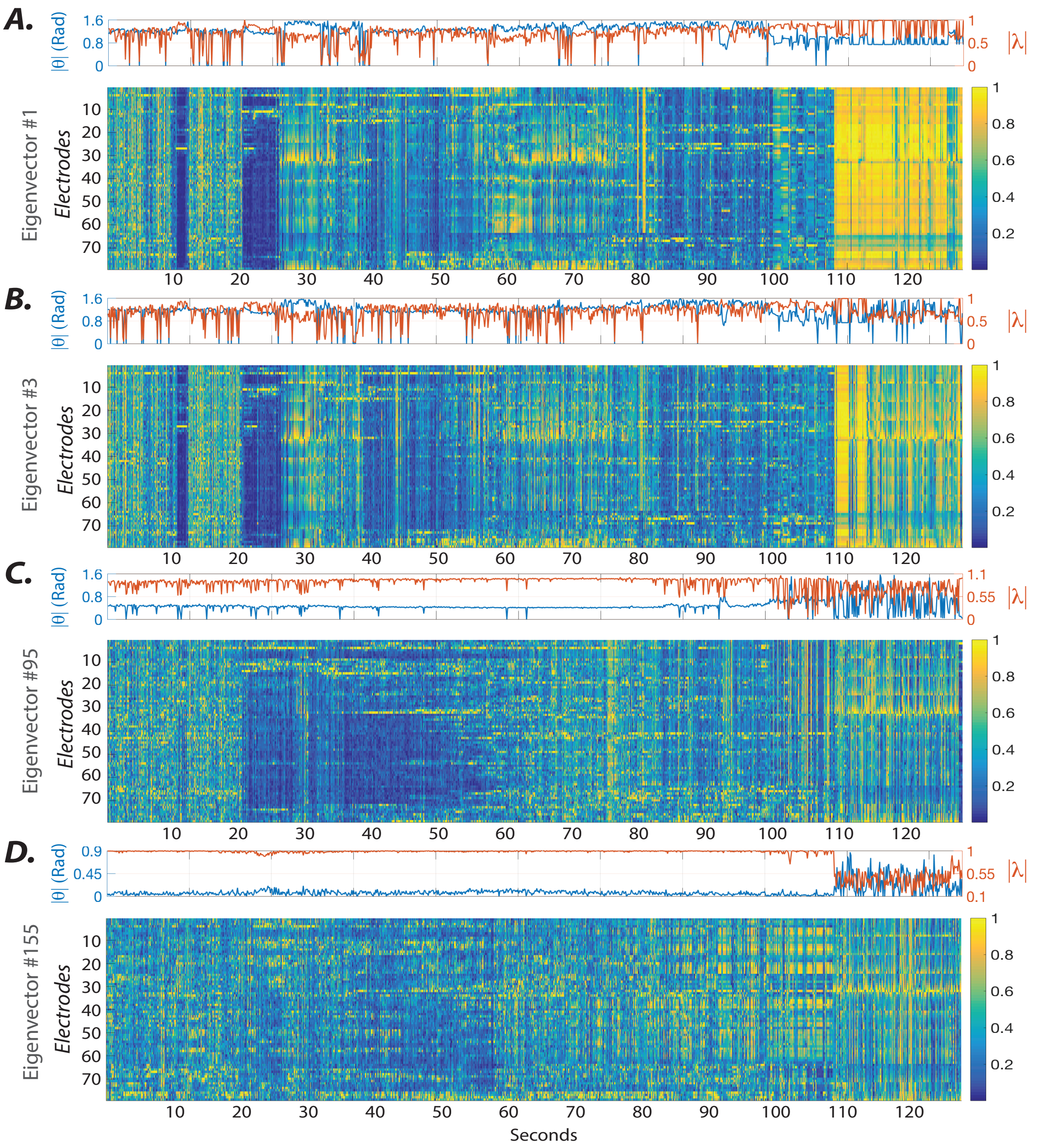

Figure 5: **Evolution of eigenmodes estimated from a second-order autoregressive model over the ictal period shown in Fig. 2A**. **(A-D)** The frequency (cyan trace) and stability (red trace) of eigenvalues associated with four representative eigenvectors – two associated with high frequencies and two associated with low frequencies – whose evolution is displayed in heatmaps. Note that the second-order model captures the emergence of local ictal sources following the seizure onset similar to the first-order model in Fig. 2.

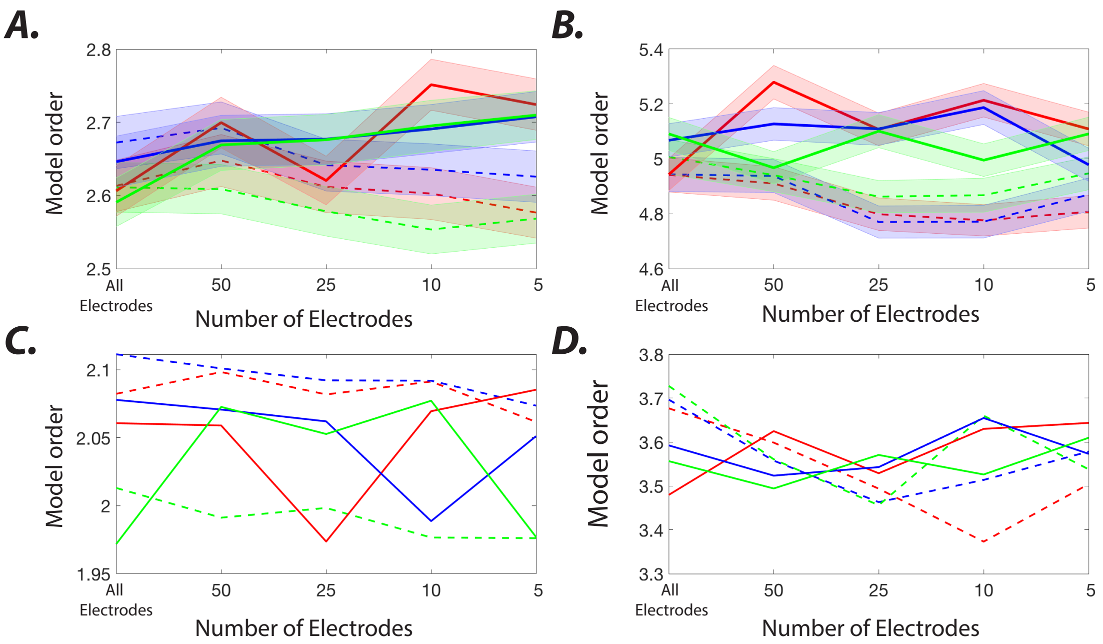

Figure 6: Estimated order of the AR model. (**A**) The shaded lines show the mean and standard error of the AR model order estimated using Schwarz's Bayesian Criterion from a 5-second window before (dashed line) and after (solid lines) the unequivocal onset of seizures from localizing patients. The x-axis shows the number of randomly sampled electrodes from each patient, used for calculating the order of the AR model. The red lines show the average order calculated from the original time series, and the blue and green lines show the results from the down-sampled time series at a half and a quarter of the original sampling rates, respectively. (**B**) The shaded lines show the mean and standard error of the AR model order estimated using Akaike information criterion from a 5-second window before (dashed line) and after (solid lines) the unequivocal onset of seizures from localizing patients. (**C&D**) The lines show the standard deviation of the AR model order estimated using Schwarz's Bayesian Criterion (**C**) and Akaike information criterion (**D**) from a 5-second window before (dashed line) and after (solid lines) the unequivocal onset of seizures from localizing patients.

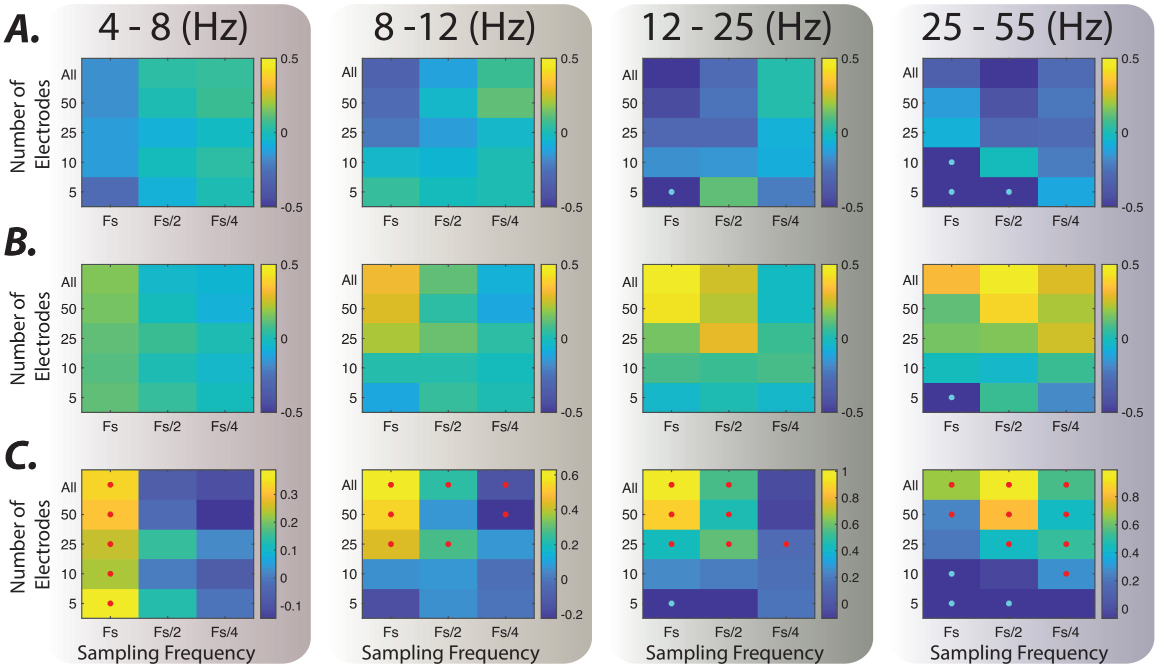

Figure 7: The wide-band increase in the kurtosis of the estimated eigenvectors following seizure onset in the down-sampled time series. (A&B) Panels show the average normalized (*z*-score) kurtosis calculated from eigenvectors associated with different frequency bands from a 5-second window before (**A**) and after (**B**) the unequivocal onset of seizures across all localizing samples. The y-axis shows the number of randomly sampled electrodes from each patient, used for estimating the first-order AR model. The y-axis shows the sampling rate; the first column shows the results calculated from the original time series (Fs), and the second and third column shows the results from the down-sampled time series at a half (Fs/2) and a quarter (Fs/4) of the original sampling rates, respectively. The elements marked by the cyan '*' show the instances where no eigenmode was found in that frequency bands across all samples. (**C**) Panels show the difference between the results in panels **B** and **A**. The elements marked by the red '*' show the instances with significant change from the period before to immediately after seizure onset (significance is evaluated by a non-parametric Wilcoxon rank-sum test, $p < 0.05$, corrected for multiple comparisons using Bonferroni correction). Note that changes in the kurtosis capture the emergence of ictal oscillations across different choices of spatiotemporal downsampling. These results also show that the modeled system is influenced by the sampling rate, the spatial coverage of seizure sources, and the total number of electrodes.

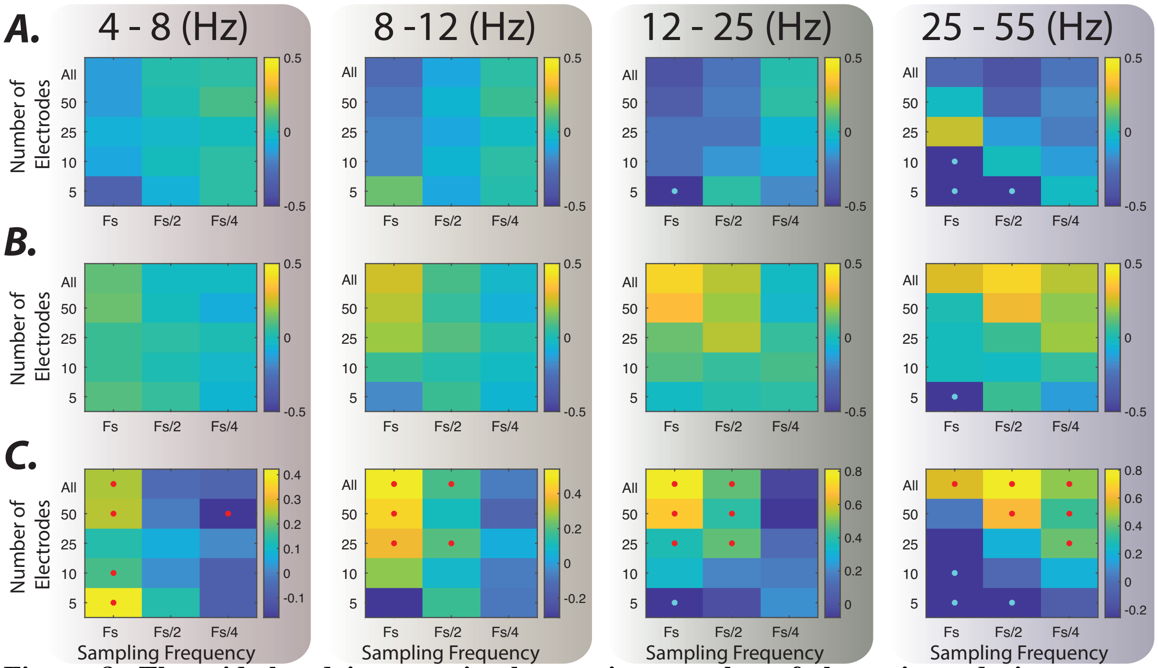

Figure 8: The wide-band increase in the maximum value of the estimated eigenvectors following seizure onset in the down-sampled time series. (**A&B**) Panels show the average normalized (*z*-score) maximum value calculated from eigenvectors associated with different frequency bands from a 5-second window before (**A**) and after (**B**) the unequivocal onset of seizures across all localizing samples. The y-axis shows the number of randomly sampled electrodes from each patient, used for estimating the first-order AR model. The y-axis shows the sampling rate; the first column shows the results calculated from the original time series (Fs), and the second and third column shows the results from the down-sampled time series at a half (Fs/2) and a quarter (Fs/4) of the original sampling rates, respectively. The elements marked by the cyan '*' show the instances where no eigenmode was found in that frequency bands across all samples. (**C**) Panels show the difference between the results in panels **B** and **A**. The elements marked by the red '*' show the instances with significant change from the period before to immediately after seizure onset (significance is evaluated by a non-parametric Wilcoxon rank-sum test, $p < 0.05$, corrected for multiple comparisons using Bonferroni correction). Note that changes in the maximum value capture the emergence of ictal oscillations across different choices of spatiotemporal downsampling. These results also show that the modeled system is influenced by the sampling rate, the spatial coverage of seizure sources, and the total number of electrodes.

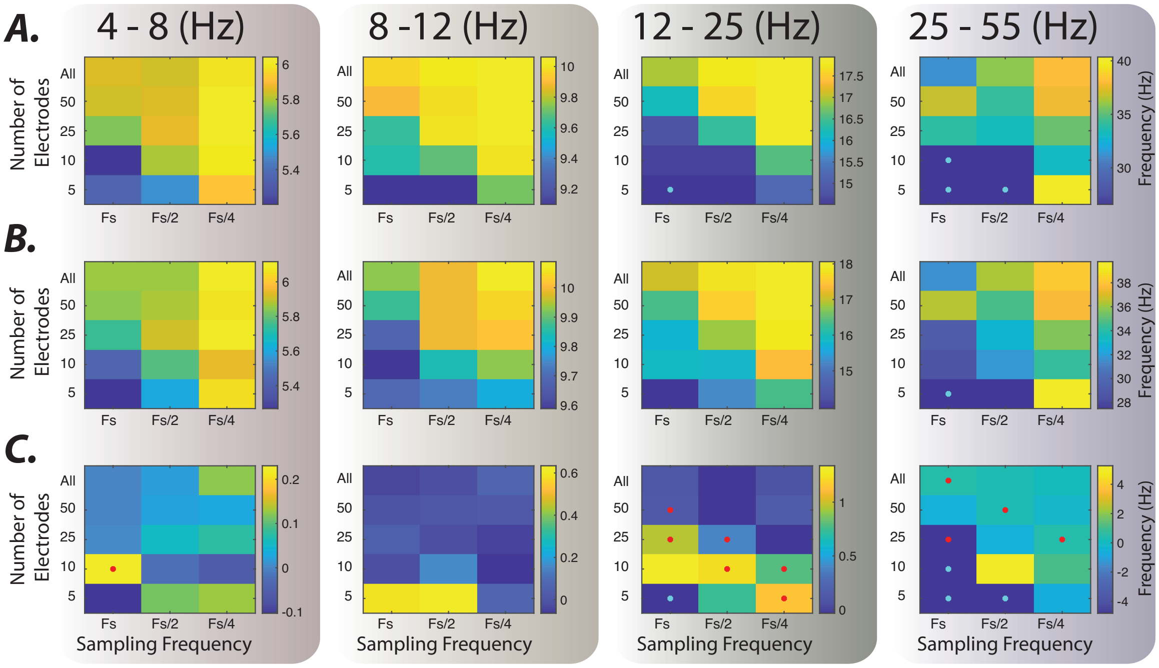

Figure 9: The effect of spatial and temporal downsampling on the frequency of the eigenmodes of the modeled first-order system. (A&B) Panels show the average frequency calculated from the eigenvalues associated with different frequency bands from a 5-second window before (**A**) and after (**B**) the unequivocal onset of seizures across all localizing samples. The y-axis shows the number of randomly sampled electrodes from each patient, used for estimating the first-order AR model. The y-axis shows the sampling rate; the first column shows the results calculated from the original time series (Fs), and the second and third column shows the results from the down-sampled time series at a half (Fs/2) and a quarter (Fs/4) of the original sampling rates, respectively. The elements marked by the cyan '*' show the instances where no eigenmode was found in that frequency bands across all samples. Overall, these results show that the spectral profile of the modeled system, especially in the gamma band (25-55 Hz), is influenced by the sampling rate, the spatial coverage of seizure sources, and the total number of electrodes. (**C**) Panels show the difference between the results in panels **B** and **A**. The elements marked by the red '*' show the instances with significant change from the period before to immediately after seizure onset (significance is evaluated by a non-parametric Wilcoxon rank-sum test, $p < 0.05$, corrected for multiple comparisons using Bonferroni correction). Note that although we identify significant changes in the average frequency of high-frequency oscillations (25-55 Hz), overall the eigenmodes' average frequency is not a sensitive marker of the emerging ictal oscillations as it is dependent on the spatiotemporal sampling.

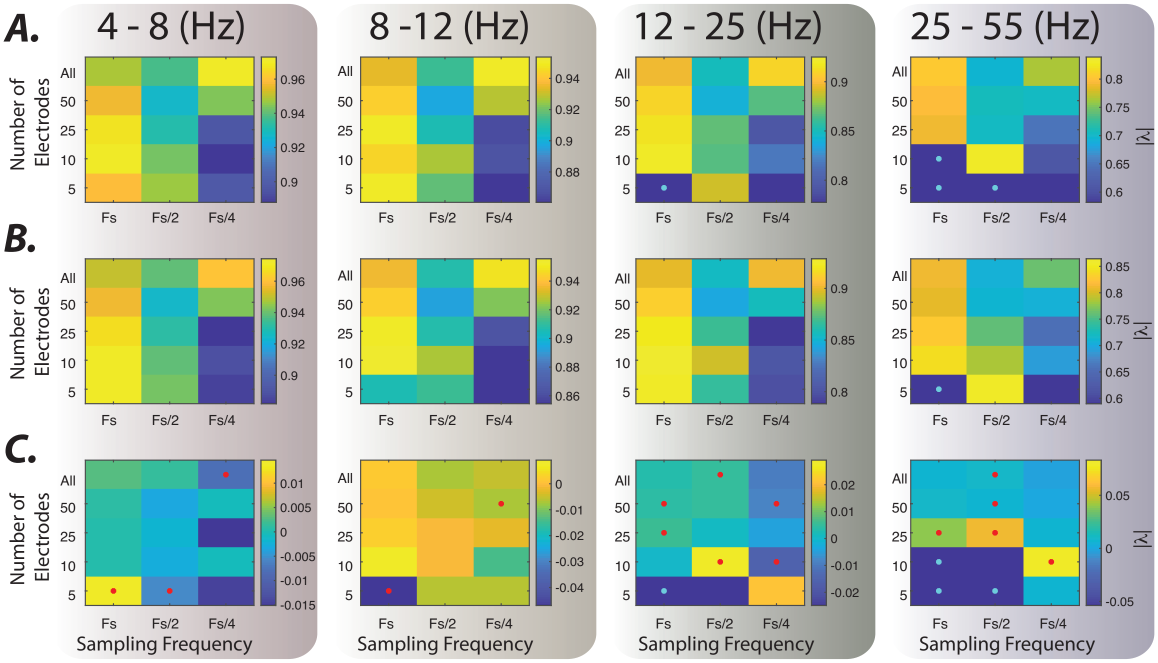

Figure 10: The effect of spatial and temporal downsampling on the stability of the eigenmodes of the modeled first-order system. (A&B) Panels show the average stability values (i.e., |λ|) calculated from the eigenvalues associated with different frequency bands from a 5-second window before (**A**) and after (**B**) the unequivocal onset of seizures across all localizing samples. The y-axis shows the number of randomly sampled electrodes from each patient, used for estimating the first-order AR model. The y-axis shows the sampling rate; the first column shows the results calculated from the original time series (Fs), and the second and third column shows the results from the down-sampled time series at a half (Fs/2) and a quarter (Fs/4) of the original sampling rates, respectively. The elements marked by the cyan '*' show the instances where no eigenmode was found in that frequency bands across all samples. Overall, these results demonstrate that the stability profile of the modeled system, especially in the gamma band (25-55 Hz), is influenced by the sampling rate, the spatial coverage of seizure sources, and the total number of electrodes. (**C**) Panels show the difference between the results in panels **B** and **A**. The elements marked by the red '*' show the instances with significant change from the period before to immediately after seizure onset (significance is evaluated by a non-parametric Wilcoxon rank-sum test, $p < 0.05$, corrected for multiple comparisons using Bonferroni correction). Note that although we identify significant changes in the average frequency of high-frequency oscillations (25-55 Hz), overall the eigenmodes' average stability value is not a sensitive marker of the emerging ictal oscillations as it is dependent on the spatiotemporal sampling.

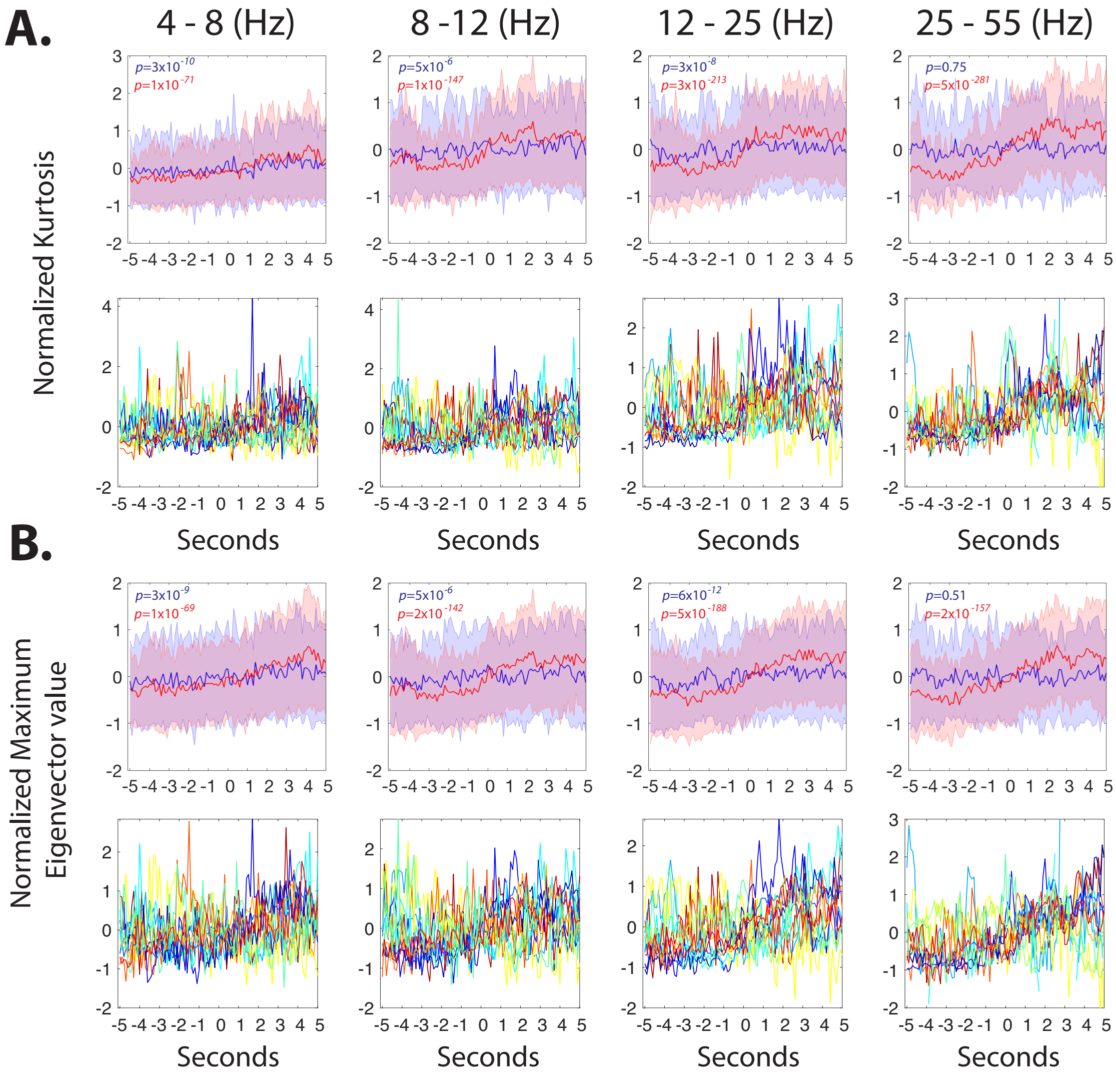

Figure 11: Wide-band increases in the maximum value and kurtosis of the estimated eigenvectors following seizure onset in non-localizing seizures. (A) Top panels show the average normalized kurtosis calculated from eigenvectors associated with different frequency bands at the earliest electrographic change (blue line) and at the unequivocal seizure onset (red line) across all non-localizing samples. The x-axis represents the time from seizure onsets. The shaded area represents the standard deviation of the samples. A non-parametric Wilcoxon rank-sum test reveals that unequivocal seizure onset is marked by a wide-band significant increase ($p < 0.05$) in the normalized kurtosis values concatenated across all seizure samples over a 10-second peri-ictal time window. The color-coded curves in the bottom panels show the average normalized kurtosis calculated for each patient separately following the unequivocal seizure onset. **(B)** The top panels show the average normalized maximum values calculated from eigenvectors associated with different frequency bands at the earliest electrographic change (blue line) and the unequivocal seizure onset (red line) across all non-localizing samples. The shaded area represents the standard deviation of the samples. Similar to panel **A**, we used a non-parametric Wilcoxon rank-sum test and observed that the unequivocal seizure onset is marked by a wide-band significant increase ($p < 0.05$) in the normalized kurtosis values concatenated across all seizure samples over a 10-second peri-ictal time window. The color-coded curves in the bottom panels show the average normalized kurtosis calculated for each patient separately

following the unequivocal seizure onset.

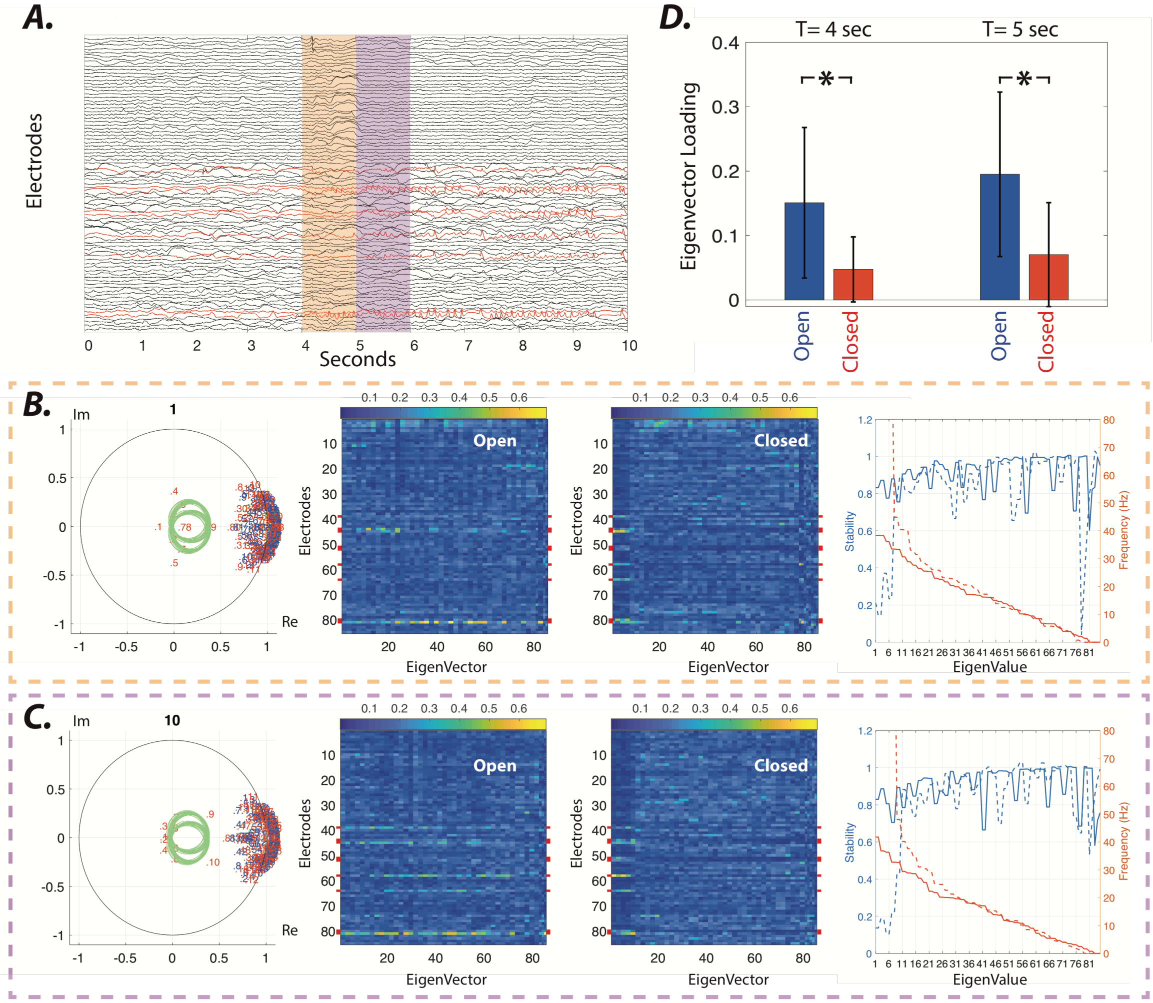

Figure 12: Stabilizing ictal onset oscillations using closed-loop feedback control for non-localizing seizures. **(A)** Seizure onset (orange window) and surrounding time periods sampled from a patient (i.e., Study 019) with iEEG recordings manifesting no clear SOZ. Electrodes that capture the emergence of ictal activity are colored red. **(B)** The left panel shows the distribution of the estimated eigenvalues at seizure onset. Eigenvalues are sorted based on their frequencies, from highest to lowest. Blue numbered dots represent the empirically estimated values. We simulate the effect of closed-loop static feedback between a few electrodes (marked red in panel **A**), by representing eigenvalues of the closed system (red numbered dots). Only nine electrodes with the highest eigenvector loading at seizure onset were selected as sensing and stimulating electrodes to mimic the limited channels available in implantable neurostimulation devices. The static output feedback gains were calculated using the generalized pole placement method [1]. The control-theoretic objective was to shift all the higher frequency (>12 Hz) eigenvalues of the system, which were previously estimated from ten consecutive sliding windows (4-5s in length) following the seizure onset to the predefined zones (represented by green circles). For further details, see the Supplementary Information. The two middle panels show the absolute values of the eigenvectors in the open and closed systems. The right panel shows *(i)* the stability as

measured by the absolute values of the eigenvalues, and *(ii)* the frequency of all eigenvalues in both the open (solid lines) and closed (dashed lines) system. Note the reduced stability of all closed system eigenvalues with associated eigenvectors manifesting high loading on stimulating electrodes (eigenmodes 1-10, marked by red in the middle panels). **(C)** Results of the analysis presented in panel **D** except this time calculated for the purple window in panel **A**. **(D)** The eigenvector loadings of the stimulation electrodes following the ictal onset for open and closed systems**.** The blue bar represents the average loading of stimulating electrodes across higher frequency eigenvectors (>12 Hz) with high stability values (>0.6) for two-time points: the point of seizure onset and the following one-second window for the seizure sample shown in panel **A**. The error bars represent standard deviation around the mean. The red bars show the same values calculated from the closed system in panel **A**. Non-parametric statistical testing shows that the loadings of stimulating channels are significantly reduced at both time points (for n = 9 stimulating electrodes each of which has n=50 (open) or n=40 (closed) stable higher frequency eigenmodes, a Wilcoxon rank-sum test gives $p = 2.5\times10^{-74}$; for n = 9 stimulating electrodes each of which has n-50 (open) or n=40 (closed) stable higher frequency eigenmodes, a Wilcoxon rank-sum test gives $p = 2.9\times10^{-70}$, respectively). Broadly, this numerical experiment demonstrates that our proposed algorithm can calculate static feedback that stabilizes the electrographic seizure activity in non-localizing seizure samples.

Choosing the length of the time window in the dynamical stability-based approach

Here, we investigate whether the accuracy of the dynamic stability approach depends on the time window over which we estimate the model. Intuitively we might imagine that if the time window is too large, we would not be able to accurately specify short time-scale changes. Therefore, we applied dynamic stability analysis to the surrogate data windowed over a range of temporal lengths (SI -Figure 13). We found that the frequency and stability estimates did indeed depend on the length of the time window over which the model is fit. Specifically, the estimated frequency and damping of the oscillations were reduced progressively for larger window sizes, likely due to the presence of temporally overlapping patterns of oscillations during the time-windows considered for estimation.

Next, we examined the effect of window size on the modeled iEEG time series. We consider 1, 2, 5, and 10 sec time-windows while maintaining 100 msec shifts. The calculations provide similar intuitions to those that we obtained from the synthetic data: we observe a loss of sensitivity to high-frequency dynamics as the length of the time-window increases (SI-Figure 14). These results provide evidence in support of smaller window sizes. Therefore, in our manuscript, we report the results obtained via 1 sec windows.

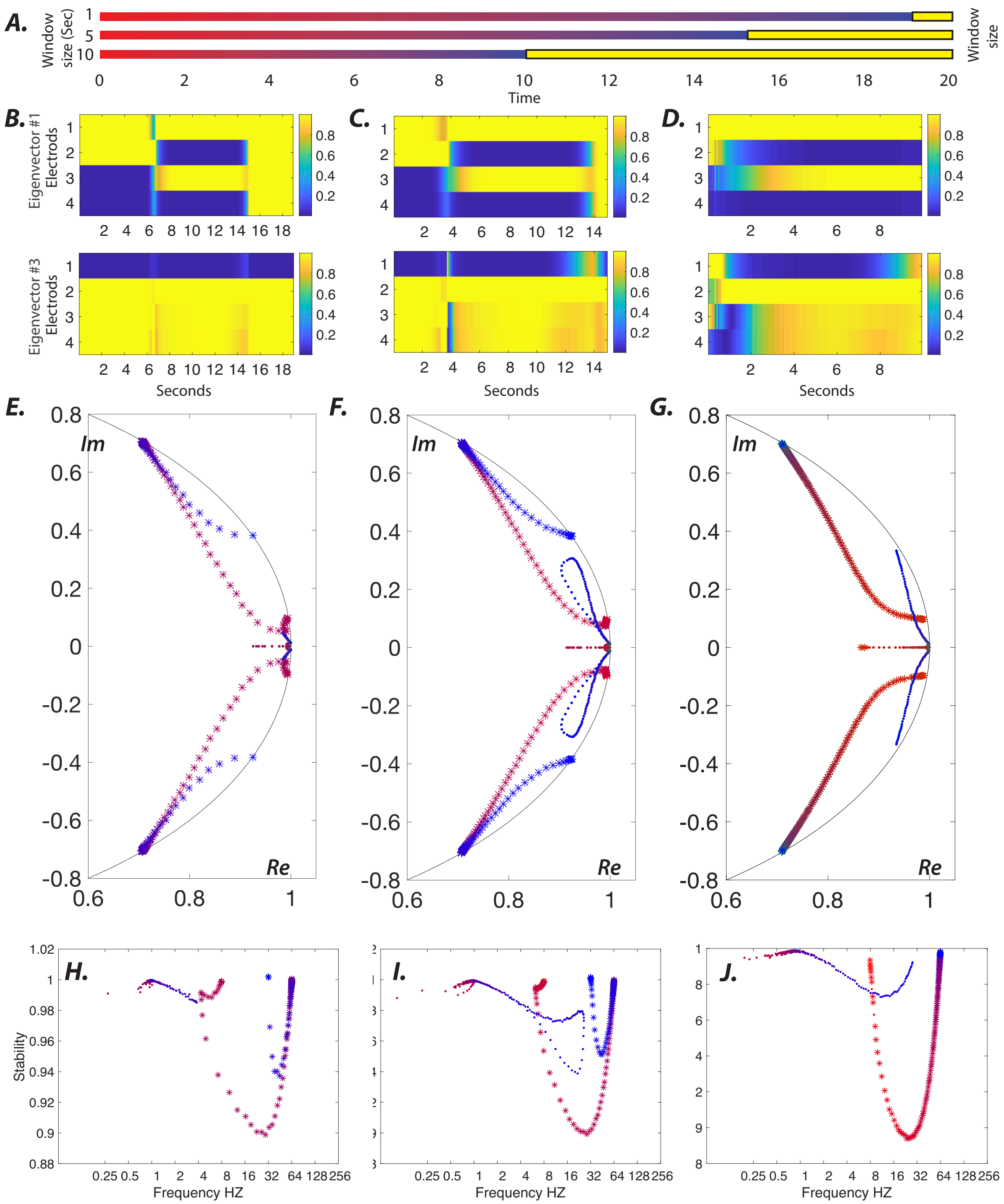

Figure 13 Effect of window size on the dynamical stability-based characterization of synthetic time-series. **(A)** Different sliding windows for the synthetic data shown in Figure 1.A in the main text are encoded by the yellow box, and the corresponding time progression is color-coded as in Figure 1.F in the main text. **(B-D)** The absolute values of the normalized first and third eigenvectors obtained using a window size of 1, 5, and 10 sec duration moved by 100 msec shifts. **(E-G)** The Argand diagram of the temporal evolution of the four eigenvalues for 1, 5, and 10 sec windows, respectively. **(H-J)** The temporal evolution of the frequency (Hz) and stability

associated with the first eigenmodes ('dots') and third eigenmodes ('stars') associated with 1, 5, and 10 sec windows. Panels **(E-J)** use the same color-coding as in panel **(A)**.

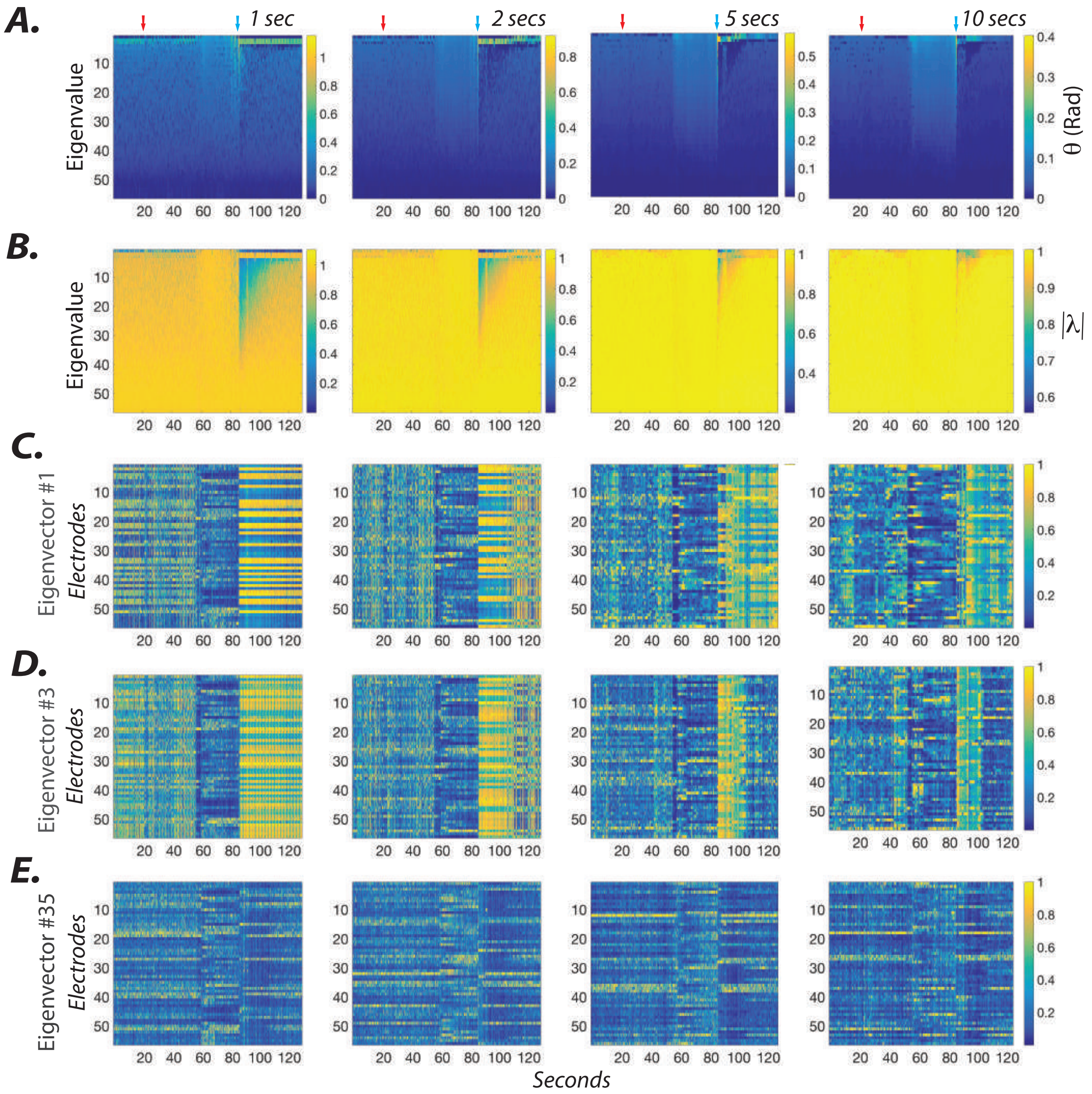

Figure 14: Effect of window size on the dynamical stability-based characterization of ictal periods. (A-B) For the example seizure from Study 020 patient, we show the time evolution of the angles and stability of the eigenvalues associated with the different eigenmodes, for different window sizes (1, 2, 5, and 10 sec; shown across different columns). The red and cyan arrows highlight the onset and offset of the ictal period, respectively. **(C-E)** The temporal evolution of three representative eigenvectors: two associated with high frequencies and one associated with low frequencies.

References

1. Pequito, S., et al., *Spectral Control of Cortical Activity*. Proceedings of the 2017 American Control Conference, 2017.
2. Gubin, L.G., B.T. Polyak, and E.V. Raik, *The method of projections for finding the common point of convex sets*. USSR Computational Mathematics and Mathematical Physics, 1967. **7**(6): p. 1-24
3. Combettes, P.L. and H.J. Trussell, *Method of successive projections for finding a common point of sets in metric spaces*. Journal of optimization theory and applications, 1990. **67**(3): p. 487-507
4. Yang, K. and R. Orsi, *Generalized pole placement via static output feedback: A methodology based on projections*. Automatica, 2006. **42**(12): p. 2143-2150
5. Franaszczuk, P.J. and G.K. Bergey, *An autoregressive method for the measurement of synchronization of interictal and ictal EEG signals*. Biological cybernetics, 1999. **81**(1): p. 3-9.
6. Franaszczuk, P.J., G.K. Bergey, and M.J. Kamiński, *Analysis of mesial temporal seizure onset and propagation using the directed transfer function method*. Electroencephalography and clinical neurophysiology, 1994. **91**(6): p. 413-427.
7. Kautsky, J., N.K. Nichols, and P. Van Dooren, *Robust pole assignment in linear state feedback*. International Journal of control, 1985. **41**(5): p. 1129-1155
